# Isocitrate protects *DJ-1* null dopaminergic cells from oxidative stress through NADP^+^-dependent isocitrate dehydrogenase (IDH)

**DOI:** 10.1371/journal.pgen.1006975

**Published:** 2017-08-21

**Authors:** Jinsung Yang, Min Ju Kim, Woongchang Yoon, Eun Young Kim, Hyunjin Kim, Yoonjeong Lee, Boram Min, Kyung Shin Kang, Jin H. Son, Hwan Tae Park, Jongkyeong Chung, Hyongjong Koh

**Affiliations:** 1 National Creative Research Initiatives Center for Energy Homeostasis Regulation, School of Biological Sciences and Institute of Molecular Biology and Genetics, Seoul National University, Seoul, Republic of Korea; 2 Department of Pharmacology, Peripheral Neuropathy Research Center (PNRC), Dong-A University College of Medicine, Busan, Republic of Korea; 3 Graduate School of Pharmaceutical Sciences, College of Pharmacy, Ewha Womans University, Seoul, Republic of Korea; 4 Department of Physiology, Peripheral Neuropathy Research Center (PNRC), Dong-A University College of Medicine, Busan, Republic of Korea; Stanford University School of Medicine, UNITED STATES

## Abstract

*DJ-1* is one of the causative genes for early onset familiar Parkinson’s disease (PD) and is also considered to influence the pathogenesis of sporadic PD. DJ-1 has various physiological functions which converge on controlling intracellular reactive oxygen species (ROS) levels. In RNA-sequencing analyses searching for novel anti-oxidant genes downstream of DJ-1, a gene encoding NADP^+^-dependent isocitrate dehydrogenase (IDH), which converts isocitrate into α-ketoglutarate, was detected. Loss of *IDH* induced hyper-sensitivity to oxidative stress accompanying age-dependent mitochondrial defects and dopaminergic (DA) neuron degeneration in *Drosophila*, indicating its critical roles in maintaining mitochondrial integrity and DA neuron survival. Further genetic analysis suggested that DJ-1 controls IDH gene expression through nuclear factor-E2-related factor2 (Nrf2). Using *Drosophila* and mammalian DA models, we found that IDH suppresses intracellular and mitochondrial ROS level and subsequent DA neuron loss downstream of DJ-1. Consistently, trimethyl isocitrate (TIC), a cell permeable isocitrate, protected mammalian *DJ-1* null DA cells from oxidative stress in an IDH-dependent manner. These results suggest that isocitrate and its derivatives are novel treatments for PD associated with *DJ-1* dysfunction.

## Author summary

The molecular pathogenesis of Parkinson’s disease (PD) is still elusive even though many causative genes for the disease have been identified. In this study, we demonstrated that isocitrate dehydrogenase (IDH), the enzyme responsible for converting isocitrate into α-ketoglutarate, is critical for the pathogenesis of PD by providing NADPH as a reducing power in the cell. *IDH* mutant animals showed increased reactive oxygen species (ROS) levels and phenotypes related to PD including dopaminergic (DA) neuron degeneration and locomotor defects. Conversely, elevating IDH function either by overexpression or treating a cell-permeable derivative of isocitrate, trimethyl isocitrate (TIC), made DA cells resist oxidative stress and reduce ROS level, thereby suppressing PD phenotypes induced by *DJ-1* mutations. These results demonstrate that IDH protects DA neurons from ROS at the downstream of DJ-1 and cell-permeable isocitrates can be novel treatments for PD.

## Introduction

Parkinson’s disease (PD) is the second most common neurodegenerative disease and is characterized by typical movement disorders and selective loss of dopaminergic (DA) neurons in the substantia nigra pars compacta (SNpc) [1]. Accumulated evidence has firmly linked the death of these neurons to oxidative stress, the state of imbalance between generation and elimination of reactive oxygen species (ROS) [2]. Postmortem brain analysis showed that markers of oxidative damage to lipids, proteins, and nucleic acids are substantially elevated in the SNpc of PD patients [2]. High levels of somatic mitochondrial DNA (mtDNA) deletion are also found in the SNpc neurons from PD patients [3], suggesting a vicious cycle of oxidative damage to mtDNA and other mitochondrial components, thus increasing ROS production in the course of DA neuron degeneration. The link between oxidative stress and DA neuronal loss is further supported by modeling parkinsonism in various animals using oxidative stress-inducing agents, such as 1-methyl-4-phenyl-1,2,3,6-tetrahydropyridine (MPTP), rotenone, paraquat, and 6-hydroxydopamine (6-OHDA) [4–8]. In addition to PD, other neurodegenerative diseases including Alzheimer’s disease (AD), Huntington’s disease (HD), and amyotrophic lateral sclerosis (ALS) are also associated with oxidative stress, further strengthening the correlation between oxidative stress and neurodegeneration [9]. However, the molecular mechanisms resulting in DA neuron degeneration under oxidative stress have not been fully elucidated.

Although PD mainly occurs in a sporadic manner, it could also occur by monogenic mutations [10]. Because familial forms of PD are often clinically and pathologically indistinguishable from sporadic ones, they are likely to have common pathogenic mechanisms [11]. Moreover, recent genome-wide association studies (GWAS) have revealed variations in several familial PD genes as significant risk factors for the development of sporadic PD [12]. Therefore, investigating how PD gene mutations cause familial PD could potentially reveal the molecular pathogenesis of sporadic PD.

Among PD-linked genes, *DJ-1* is most closely associated with oxidative stress [2]. *DJ-1* was first identified as an oncogene that transforms mouse NIH3T3 cells in cooperation with activated Ras [13]. Later, Bonifati et al. found that *DJ-1* is associated with an autosomal recessive early onset type of familial PD [14]. *DJ-1*-deficient animal models showed hypersensitivity to oxidative stress [15–18], and further cell biological studies revealed that DJ-1 is a multifunctional protein that participates in transcription regulation, anti-apoptotic signaling, protein stabilization and degradation, and mitochondrial regulation to respond to oxidative stress [19]. DJ-1 is sequentially oxidized on its cysteine residues, and its activity and subcellular localization are regulated by its oxidative status [20–23]. Excessive oxidation of DJ-1 inactivates it, and this oxidized form is observed in the brains of patients with sporadic PD and AD [24, 25], suggesting that DJ-1 participates in the pathogenesis of sporadic PD as well as familial PD. In *Drosophila*, there are two homologues of mammalian *DJ-1*; *DJ-1α* and *β*. *DJ-1α* is predominantly expressed in the testes, whereas *DJ-1β* is expressed throughout the whole body [16, 18, 26], similar to the expression pattern of mammalian *DJ-1* [13]. *DJ-1β* loss-of-function mutants show locomotive dysfunction and loss of DA neurons, resembling the phenotypes seen in PD patients [18, 27].

In this study, we found that DJ-1 is critical for maintaining transcription of NADP^+^-dependent isocitrate dehydrogenase (IDH) under oxidative stress induced by pesticides like rotenone that have been associated with onset of PD in recent epidemiologic studies [28]. IDH catalyzes decarboxylation of isocitrate into α-ketoglutarate and CO_2_, and also produces NADPH, which provides a reducing power to antioxidant processes scavenging ROS [29]. Indeed, our *Drosophila IDH* mutants showed decreased NADPH levels with increased ROS production and hyper-sensitivity to oxidative stress. Moreover, loss of *IDH* induced age-dependent mitochondrial defects and DA neuron degeneration, very similar to the phenotypes of *Drosophila* PD models [30]. Consistently, overexpression of *IDH* in *DJ-1* mutants successfully enhanced their survival rates and ameliorated DA neuron loss under oxidative stress. Further genetic analysis revealed that DJ-1 maintains *IDH* expression by regulating the Kelch-like ECH-associating protein 1 (Keap1)-nuclear factor-E2-related factor2 (Nrf2) pathway. In addition, trimethyl isocitrate (TIC), a cell permeable form of isocitrate, markedly restored oxidative stress-induced decrease of NADPH level and inhibited subsequent cell death in mammalian DA cells with *DJ-1* deficiency. These results consistently support that the activity of NADP^+^-dependent IDH is critical in protecting neurons from oxidative stress and *DJ-1* mutation.

## Results

### RNA-sequencing analysis reveals defected *IDH* expression in *DJ-1β* mutant flies under oxidative stress

**Fig 1:**
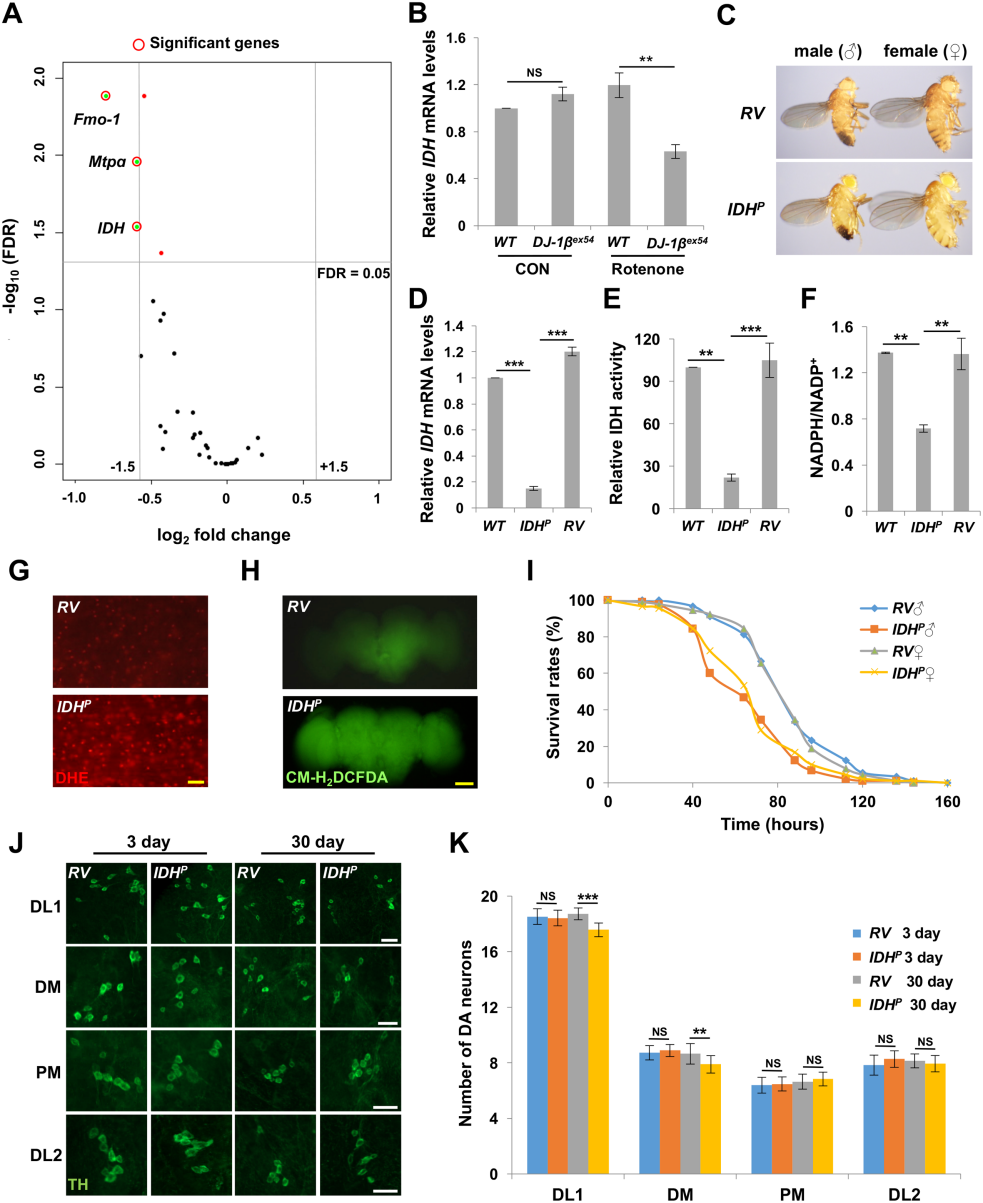
IDH suppresses oxidative stress and DA neuron loss in *Drosophila*. (A) The volcano plot from RNA-seq data presents the value of fold change and false discovery rate (FDR) of the 34 genes of which gene expression was reduced in *DJ-1ß* null mutants under oxidative stress. Vertical lines (gray) indicate fold change = ±1.5. Horizontal line (gray) indicates FDR = 0.05. (B) Comparison of *IDH* mRNA expression levels in the heads and thoraces of wild type flies (*WT*) and *DJ-1β* null mutants (*DJ-1β*^*ex54*^) under control (CON) or rotenone treatment (Rotenone) (n = 3). (C) Light stereo micrographs of revertant (*RV*) and *IDH*^*P*^ flies. (D-F) Comparison of *IDH* mRNA levels (D), IDH activity (E), and NADPH/NADP^+^ ratio (F) in *WT*, *IDH*^*P*^ and *RV* flies (n = 3). (G) DHE staining of the indirect flight muscle from fly thoraces. Scale bars: 10 μm. (H) CM-H_2_DCFDA staining of fly brains. Scale bars: 100 μm. (I) Survival curves of *RV* and *IDH*^*P*^ mutant male (♂) and female (♀) flies under rotenone treatments (log-rank test: P<0.001, n = 90 for each genotype). All life span assays were carried out at 25°C and were repeated at least twice. For detailed statistical analysis of life span data in this and other figures, please see S3 Table. (J) Confocal images of DA neurons within DL1, DM, PM, and DL2 clusters of the adult brains from 3- and 30-day-old *RV* controls and *IDH*^*P*^ mutants on normal media. DA neurons were stained with anti-TH antibody (green). Scale bars: 20 μm. (K) Graphs of the average number of DA neurons in each cluster (n = 36). Data information: Significance was determined by one-way ANOVA with Sidak correction [**, P<0.01; ***, P<0.001; NS, not significant (P>0.05)]. Error bars indicate SD. Details of all the indicated genotypes in this and other figures are described in Materials and Methods.

To find out a new molecular mechanism in which DJ-1 protects cells from oxidative stress, we treated rotenone, a well-known ROS inducer associated with PD [28], to wild type and *DJ-1β*-deficient flies, and investigated gene expression in both of them through RNA-sequencing (RNA-seq) analysis. Based on the role of mitochondria as a center for generating and controlling ROS [9], we hypothesized that a ROS controlling protein located in mitochondria would act downstream of DJ-1. We looked over the RNA-seq result and found that oxidation-reduction process gene ontology was the most changed in biological process terms (S1 Table) and oxidoreductase activity gene ontology was the most changed in molecular function terms (S2 Table) between wild type and *DJ-1β*-deficient flies, consistent with the role of DJ-1 in oxidative stress responses. We further looked into the gene list falling into two groups: oxidation-reduction process (GO: 0016491) and mitochondrion (GO: 0005739). As a result, 173 genes in oxidation-reduction process ontology and 237 genes in mitochondrion ontology were found to be different between wild type and *DJ-1ß* null mutants in RNA-seq analysis. Interestingly, 34 genes were included in both ontologies (S1A Fig), and most of the genes diminished their mRNA expression in *DJ-1β* null mutants (S1A and S1B Fig). Among them, 3 genes showed statistically significant difference in false discovery rate (FDR) < 0.05 and satisfied fold change > 1.5 at the same time. As we expected, *IDH*, which encodes a protein regulating intracellular ROS level by producing NADPH, was one of the 3 genes (Fig 1A and Table 1). In quantitative RT-PCR, *IDH* gene expression was decreased in *DJ-1β* null mutants compared to wild type controls under oxidative stress, confirming the RNA-seq data (Fig 1B). The reduction in *IDH* expression in *DJ-1β* null mutants was observed in heads, thoraces and abdomens, indicating that it may be not tissue-specific (S1C–S1E Fig).

**Table 1:** List of differentially expressed genes in *DJ-1β* null flies on rotenone-containing media compared to wild type controls.

Flybase ID	Fly gene name	Human orthologue	Function	Absolute fold	FDR
FBgn0034943	Fmo-1	FMO1/2/3/4/5	Oxidative metabolism of a variety of xenobiotics	-1.74	0.0041549
FBgn0028479	Mtα	HADHA	Fatty acid β-oxidation	-1.51	0.0110128
FBgn0001248	IDH	IDH1/2	Catalyzing the oxidative decarboxylation of isocitrate to α-ketoglutarate	-1.51	0.0290009

In mammalian organisms, IDH1 and IDH2 are located in cytosol and mitochondria, respectively, and they are expressed from independent genes [29]. However, in *Drosophila*, a cytosolic isoform (IDHc) and mitochondrial isoforms (IDHm1 and IDHm2) are expressed from the single gene *IDH*, although *Drosophila* IDHs are highly homologous to human counterparts (S2A and S2B Fig). *IDH*^*P*^ is a *Drosophila* mutant line containing a P-element in an exon that is shared by all isoforms of *IDH* (S2C Fig). *IDH*^*P*^ flies successfully developed into adults (Fig 1C), but they showed decreased *IDH* mRNA expression, IDH enzyme activity, and NADPH/NADP^+^ ratio compared to wild type controls (Fig 1D–1F). All these phenotypes were rescued in the revertant (*RV*) generated by precise P-element excision (Fig 1D–1F). These results demonstrated that the P-element insertion successfully inhibits gene expression, activity, and function of IDH in the *IDH* mutants. In addition, the inserted P-element slightly increased expression of *CG17352*, a gene located next to *IDH* (S2C and S2D Fig). However, compared to control flies, *CG17352* transgenic flies showed no difference in survival rates under oxidative stress (S2E Fig), ruling out the possibility that this small increase of *CG17352* expression affects the oxidative stress-related phenotypes of *IDH*^*P*^ mutants that we examined in following experiments.

### *IDH* mutants show age-dependent mitochondrial and DA neuronal defects

To understand physiological functions of IDH, we checked the lifespan of *IDH*^*P*^ flies that exhibited notably shorter life spans than *WT* and *RV* controls (S2F Fig). Furthermore, ROS markers such as dihydroethidium (DHE) and 5-(and-6)-chloromethyl-2′,7′-dichlorodihydrofluorescein diacetate (CM-H_2_DCFDA) demonstrated that *in vivo* ROS accumulated highly in *IDH* mutants (Fig 1G and 1H). This finding was further confirmed by measuring the level of *hsp22* mRNA expression which increases according to mitochondrial oxidative stress (S2G Fig) [31]. Consistently, the survival rate of *IDH*^*P*^ flies radically declined under rotenone treatments showing that IDH is indispensable for sustaining resistance to oxidative stress (Fig 1I). To further investigate the effect of the *in vivo* ROS accumulation, the phenotypes of 3- and 30-day-old *IDH*^*P*^ adult flies were compared to those of *RV* control flies without oxidative insults. While ATP level in the muscles, which indicates the function of mitochondria, and mtDNA content, which shows the amount of mitochondria, did not present any significant changes in 3-day-old flies, those of 30-day-old *IDH*^*P*^ mutants were markedly dropped compared to *RV* control flies (S3A–S3D Fig). Based on the fact that oxidative stress and mitochondrial defects are vital factors in onset and deterioration of a variety of human diseases, particularly neurodegenerative diseases including PD [2], the phenotypes related to PD in *IDH* mutants were analyzed. The results showed that climbing ability and the number of DA neurons of 30-day-old *IDH* mutants substantially decreased (~30% for climbing ability, ~10% for DA neuron number) compared to those of *RV* control flies (Fig 1J and 1K, and S3E and S3F Fig). Specifically, among the four major DA neuron clusters [dorsolateral clusters 1 (DL1), dorsomedial clusters (DM), posteriomedial clusters (PM), and dorsolateral clusters 1 (DL2)] [32], only the DA neurons in DL1 and DM clusters were degenerated (Fig 1J and 1K), as previously shown in *PINK1* and *Parkin* mutant flies [32]. These results showed that DA neuronal degeneration induced by mutating *IDH* gene can be clearly distinguished from non-specific neuronal degeneration caused by simply increasing ROS.

### IDH restores the oxidative stress-induced phenotypes of *DJ-1β* mutant flies

**Fig 2:**
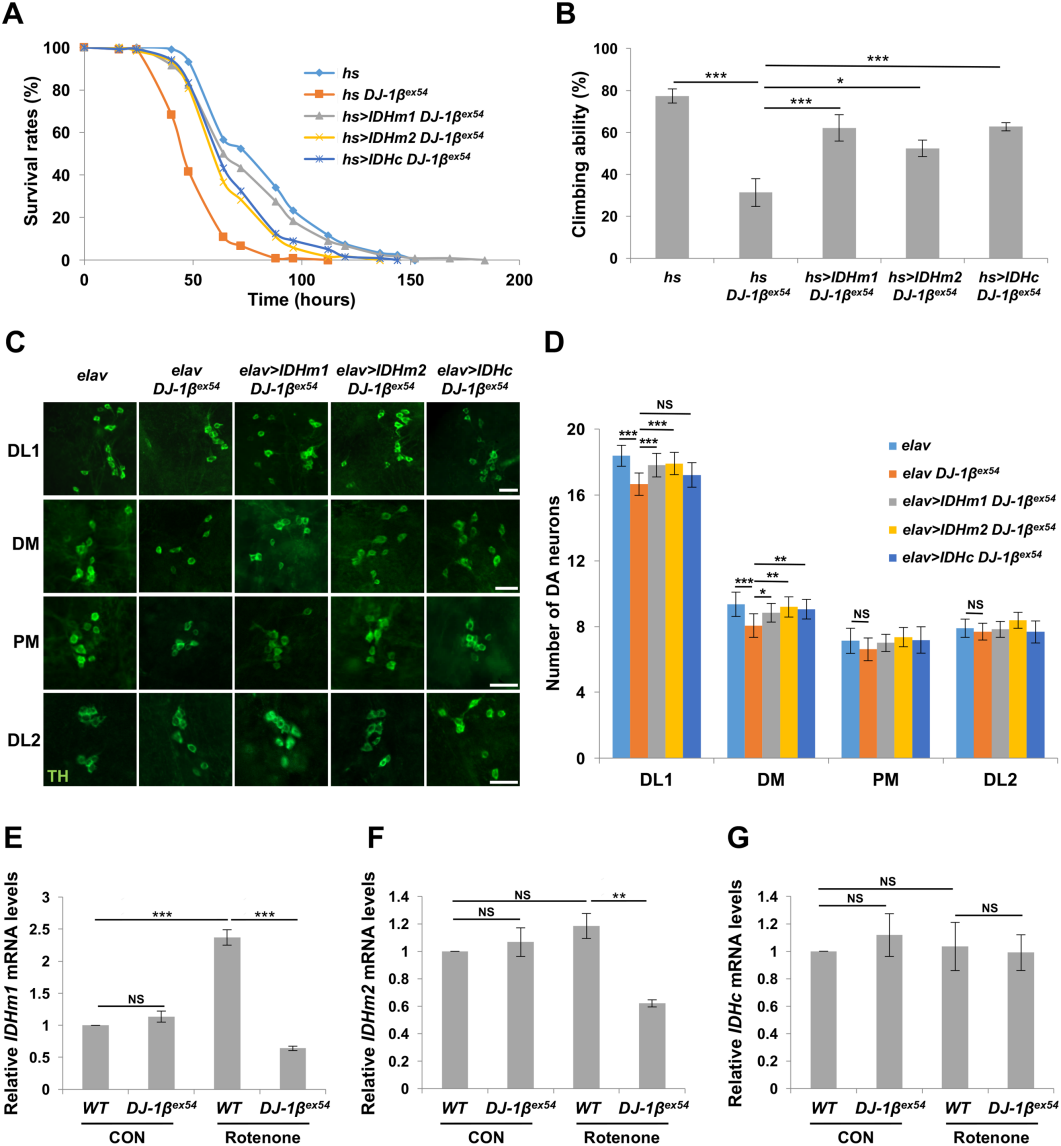
IDH rescues neuronal defects of *DJ-1* mutants under oxidative stress. (A) Survival curves of *DJ-1β* null mutants (*hs DJ-1β*^*ex54*^), IDHm1-overexpressing *DJ-1β* null mutants (*hs>IDHm1 DJ-1β*^*ex54*^), IDHm2-overexpressing *DJ-1β* null mutants (*hs>IDHm2 DJ-1β*^*ex54*^), and IDHc-overexpressing *DJ-1β* null mutants (*hs>IDHc DJ-1β*^*ex54*^) under rotenone treatment. *heat shock* (*hs*)-GAL4/+ (*hs*) flies were used as controls (log-rank test: P<0.001, n = 120 for each genotype). All life span assays were carried out at 25°C and were repeated at least twice. (B) Comparison of climbing ability of 7-day-old flies after rotenone treatments (n = 5). (C-D) Confocal images (C) and graphs (D) of the average number of DA neurons within DL1, DM, PM, and DL2 clusters of the adult brains from 6-day-old *DJ-1β* null mutants (*elav DJ-1β*^*ex54*^), IDHm1-overexpressing *DJ-1β* null mutants (*elav>IDHm1 DJ-1β*^*ex54*^), IDHm2-overexpressing *DJ-1β* null mutants (*elav>IDHm2 DJ-1β*^*ex54*^) and IDHc-overexpressing *DJ-1β* null mutants (*elav>IDHc DJ-1β*^*ex54*^) after rotenone treatments. *elav*-GAL4/+ (*elav*) flies were used as controls (n = 40 for *elav*; n = 37 for *elav DJ-1β*^*ex54*^; n = 36 for *elav>IDHm1 DJ-1β*^*ex54*^; n = 30 for *elav>IDHm2 DJ-1β*^*ex54*^; n = 39 for *elav>IDHc DJ-1β*^*ex54*^). DA neurons were stained with anti-TH antibody (green). Scale bars: 20 μm. (E-G) Comparison of *IDHm1* (E), *IDHm2* (F), or *IDHc* (G) mRNA expression levels in the heads and thoraces of wild type (*WT*) and *DJ-1β* null mutants (*DJ-1β*^*ex54*^) under control (CON) or rotenone treatments (Rotenone) (n = 3). Data information: Significance was determined by one-way ANOVA with Sidak correction [*, P<0.05; **, P<0.01; ***, P<0.001; NS, not significant (P>0.05)]. Error bars indicate SD.

*Drosophila* phenotypes related to PD symptoms, such as loss of climbing ability and DA neuronal degeneration, were also observed in *IDH*^*P*^ flies. Consistent with these results, when IDHm1, IDHm2, and IDHc isoforms were overexpressed in *DJ-1β* mutant flies, survival rates of *DJ-1β* mutant flies increased dramatically in a rotenone-induced oxidative stress condition (Fig 2A). *IDH* overexpression rescued the reduced climbing ability caused by *DJ-1β* mutation under rotenone treatment (Fig 2B), and suppressed the DA neuronal degeneration caused by rotenone or H_2_O_2_ treatment in *DJ-1β* mutants (Fig 2C, 2D and S4 Fig). Moreover, *IDH*^*P*^ mutation decreased survival rates and the number of DA neurons under oxidative stress, but the genetic combining of *IDH*^*P*^ mutation did not have an effect on *DJ-1β* mutants (S5 Fig). From these results, we concluded that IDH is an important antioxidant enzyme that protects cells downstream of DJ-1 under oxidative stress. Especially, expressing IDHm1 and IDHm2 more effectively restored the phenotypes of *DJ-1β* mutants induced by oxidative stress (Fig 2A–2D and S4 Fig), indicating that mitochondrial IDH plays an important role in DJ-1-regulated cell protection against oxidative stress. Consistent with this idea, in *DJ-1β* deficient flies stressed with rotenone, the mRNA expression of mitochondrial IDH isoforms (IDHm1 and IDHm2) was severely decreased, but that of cytosolic IDH isoform (IDHc) showed no significant change (Fig 2E–2G). In addition, when we overexpressed IDH genes in *PINK1* mutants which showed reduced levels of ATP and mtDNA in muscles, loss of climbing ability, and DA neuronal degeneration in the absence of oxidative stress [32], none of these phenotypes were rescued (S6 Fig). These results suggested that IDH is specifically related to DJ-1 in PD pathology.

### Nrf2 pathway regulates the expression of *IDH* in a DJ-1-dependent manner

**Fig 3:**
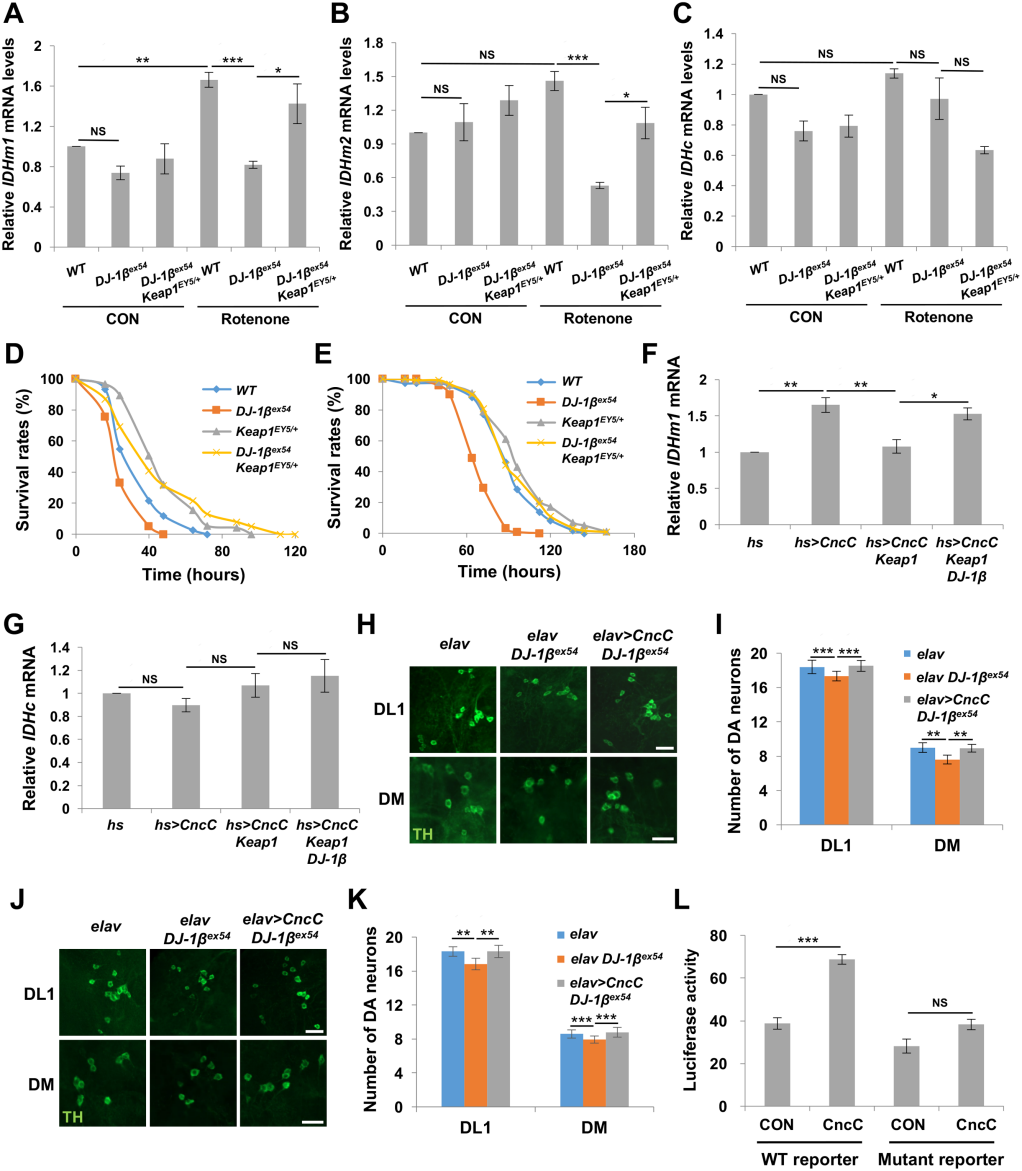
IDH mRNA expression is induced by DJ-1 through Nrf2 pathway. (A-C) Comparison of *IDHm1* (A), *IDHm2* (B), or *IDHc* (C) mRNA expression levels in the whole body of wild type (*WT*), *DJ-1β* null mutants (*DJ-1β*^*ex54*^) and *DJ-1β* null mutants with a heterozygous *Keap1* mutation (*DJ-1β*^*ex54*^ *Keap1*^*EY5/+*^) under control (CON) or rotenone treatment (Rotenone) (n≥3). (D) Survival curves of wild types (*WT*), *DJ-1β* null mutants (*DJ-1β*^*ex54*^), *Keap1* heterozygous mutants (*Keap1*^*EY5/+*^) and *DJ-1β* null mutants with a heterozygous *Keap1* mutation (*DJ-1β*^*ex54*^ *Keap1*^*EY5/+*^) under rotenone treatment (log-rank test: *DJ-1β*^*ex54*^ VS *WT* & *DJ-1β*^*ex54*^ VS *DJ-1β*^*ex54*^ *Keap1*^*EY5/+*^: P<0.001, *Keap1*^*EY5/+*^ VS *DJ-1β*^*ex54*^ *Keap1*^*EY5/+*^: P = 0.9896, n = 120 for *WT* and *DJ-1β*^*ex54*^; n = 150 for *Keap1*^*EY5/+*^; n = 140 for *DJ-1β*^*ex54*^ *Keap1*^*EY5/+*^) (E) Survival curves of adult flies under H_2_O_2_ treatment (log-rank test: *DJ-1β*^*ex54*^ VS *WT* & *DJ-1β*^*ex54*^ VS *DJ-1β*^*ex54*^ *Keap1*^*EY5/+*^: P<0.001, *Keap1*^*EY5/+*^ VS *DJ-1β*^*ex54*^ *Keap1*^*EY5/+*^: P = 0.1613, n = 109 for *WT*; n = 119 for *DJ-1β*^*ex54*^; n = 100 for *Keap1*^*EY5/+*^; n = 120 for *DJ-1β*^*ex54*^ *Keap1*^*EY5/+*^). All life span assays were carried out at 25°C and were repeated at least twice. (F) Comparison of *IDHm1* mRNA expression levels in the whole body of *hs-GAL4* control flies (*hs*), CncC-overexpressing files (*hs>CncC*), CncC and Keap1-overexpressing flies (*hs>CncC Keap1*) and CncC, Keap1 and DJ-1β-overexpressing flies (*hs>CncC Keap1 DJ-1β*) (n≥3). (G) Comparison of *IDHc* mRNA expression levels in the whole body of flies (n≥3). (H-I) Confocal images (H) and graphs (I) of the average number of DA neurons within DL1 and DM clusters of the adult brains from 6-day-old *elav-GAL4* control flies (*elav*), *DJ-1β* null mutants (*elav DJ-1β*^*ex54*^) and CncC-overexpressing *DJ-1β* null mutants (*elav>CncC DJ-1β*^*ex54*^) after rotenone treatments. DA neurons were stained with anti-TH antibody (green). (n = 30 for each genotype). Scale bars: 20 μm. (J-K) Confocal images (J) and graphs (K) of the average number of DA neurons within DL1 and DM clusters of the adult brains of the 6-day-old flies after H_2_O_2_ treatments. DA neurons were stained with anti-TH antibody (green). (n = 27 for *elav*; n = 30 for other genotypes). Scale bars: 20 μm. (L) Comparison of luciferase activity in control (Con) or CncC-transfected (CncC) S2 cells (n = 3). The reporter plasmid with wild type (*WT*) or ARE site-mutated (Mut) *IDH* promoter was co-transfected to quantitatively measure activation of each promoter by CncC transcription factor. The construction of *IDH* reporters were described in Materials and Methods and S2C Fig. Data information: Significance was determined by one-way ANOVA with Sidak correction [*, P<0.05; **, P<0.01; ***, P<0.001; NS, not significant (P>0.05)]. Error bars indicate SD.

The transcription factor Nrf2 induces expression of various antioxidant proteins in response to oxidative stress [33]. Under normal conditions, Nrf2 is degraded by Keap1, an E3 ligase. Upon exposure to oxidative stress, Nrf2 is stabilized and translocated to the nucleus [34–37]. Interestingly, DJ-1 stabilizes Nrf2 by preventing its association with Keap1 and thereby preventing the subsequent degradation [38]. Previous data showed that Nrf2 also controls *IDH* mRNA expression [39]. Thus, we suspected Nrf2 to be a possible transcription factor that mediates *IDH* mRNA expression regulated by DJ-1. When we checked mRNA levels of each IDH isoform, the expression levels of *IDHm1* and *IDHm2* were rescued by *Keap1* mutation in *DJ-1β* null flies under oxidative stress (Fig 3A and 3B). In contrast, *Keap1* mutation failed to change *IDHc* mRNA expression in the stressed *DJ-1β* mutants, suggesting that DJ-1 specifically induces mitochondrial IDH isoforms via Keap1-Nrf2 pathways (Fig 3C). In addition, *Keap1* mutation also increased survival rates of *DJ-1β* mutants on rotenone- or H_2_O_2_-containing media (Fig 3D and 3E). Consistently, Cap’n’Collar C (CncC), a fly orthologue of mammalian Nrf2 [40], could induce *IDHm1* mRNA expression (Fig 3F), but failed to change *IDHc* mRNA expression (Fig 3G). When *Keap1* was co-overexpressed, *IDHm1* mRNA expression decreased, and increased again with the addition of *DJ-1β* overexpression (Fig 3F). Moreover, when *CncC* was overexpressed in *DJ-1β* mutants, DA neuronal death induced by rotenone or H_2_O_2_ was suppressed (Fig 3H–3K). These results confirmed that DJ-1 accelerates mRNA expression of mitochondrial IDH isoforms through the Keap1-Nrf2 pathway to protect the cells from oxidative stress.

In the process of investigating the mechanism of Nrf2-induced *IDH* expression, we found that a putative Nrf2-binding element, also known as Antioxidant Response Element (ARE), (TGACGGGGC) [33] is located on the promoter region of the *IDH* gene (S2C Fig), and cloned this *IDH* promoter region in a luciferase reporter plasmid. We co-transfected *CncC* cDNA and this ARE reporter plasmid in *Drosophila* S2 cell line, and found that the promoter activity increased with *CncC* expression and decreased with ARE mutation (Fig 3L). Therefore, we concluded that DJ-1 controls *IDH* expression through Nrf2 and ARE in an evolutionarily conserved manner.

### Mammalian IDHs rescue the oxidative stress-induced defects in SN4741 DA cells lacking *DJ-1*

**Fig 4:**
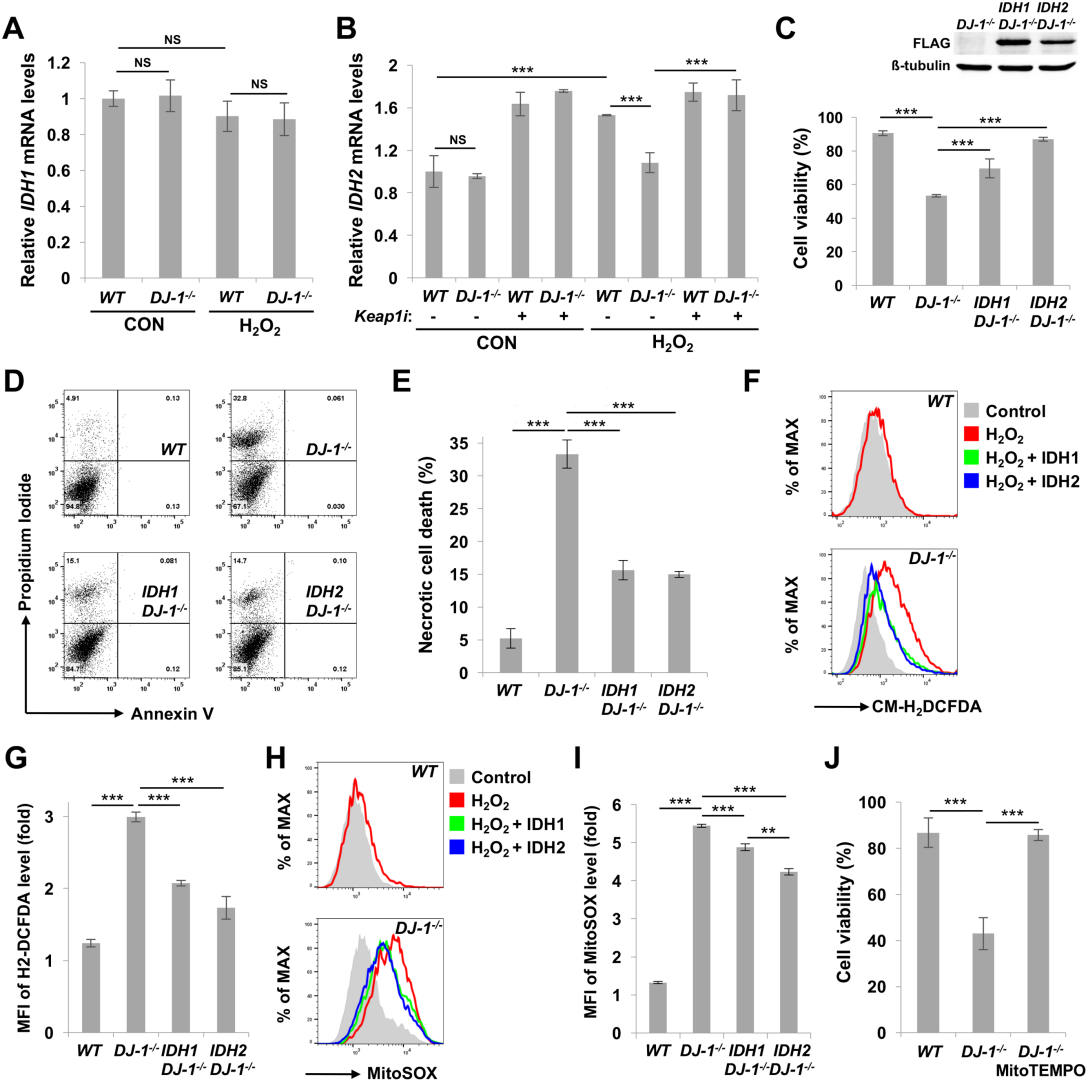
IDH inhibits oxidative stress-induced cell death in *DJ-1* null mammalian DA cells. (A) Comparison of *IDH1* mRNA expression levels in wild type (*WT*) and *DJ-1* null (*DJ-1*^*-/-*^) SN4741 cells under control (CON) or H_2_O_2_ (H_2_O_2_) treatment (n = 3). (B) *IDH2* mRNA expression level change was analyzed in *DJ-1* null SN4741 cells under control (CON) or H_2_O_2_ (H_2_O_2_) treatment, with scramble (-) or Keap1 siRNA (+) (n = 3). (C) Cell viability of wild type (*WT*), *DJ-1* null (*DJ-1*^*-/-*^), IDH1-overexpressing *DJ-1* null (*IDH1 DJ-1*^*-/-*^) and IDH2-overexpressing *DJ-1* null (*IDH2 DJ-1*^*-/-*^) SN4741 cells under 1.5 mM H_2_O_2_ treatment for 6 h. Cell viability was measured by MTT assay. The inset shows immunoblot of FLAG-tagged IDH1 and IDH2. β–tubulin as a loading control. (D) Propidium iodide and annexin V FITC staining of H_2_O_2_-treated SN4741 cells. (E) Necrotic cell death rates (n = 3). (F) Flow cytometric analysis of CM-H_2_DCFDA-stained SN4741 cells. Gray-filled area: *WT* or *DJ-1* null SN4741 cells without H_2_O_2_ treatment; Red line: H_2_O_2-_treated SN4741 cells; Green line: H_2_O_2-_treated IDH1-overexpressing SN4741 cells; Blue line: H_2_O_2-_treated IDH2-overexpressing SN4741 cells. (G) Fold change in the mean of fluorescence intensity (MFI) of CM-H_2_DCFDA in flow cytometric analysis (n = 3). (H) Flow cytometric analysis of MitoSOX-stained SN4741 cells. Gray-filled area: *WT* or *DJ-1* null SN4741 cells without H_2_O_2_ treatment; Red line: H_2_O_2_-treated SN4741 cells; Green line: H_2_O_2_-treated IDH1-overexpressing SN4741 cells; Blue line: H_2_O_2_-treated IDH2-overexpressing SN4741 cells. (I) Fold change in the mean of fluorescence intensity (MFI) of MitoSOX in flow cytometric analysis (n = 3). (J) Cell viability of MitoTEMPO-treated SN4741 cells under H_2_O_2_ treatment. Cell viability was measured by MTT assays (n = 3). Data information: Significance was determined by one-way ANOVA with Sidak correction [**, P<0.01; ***, P<0.001; NS, not significant (P>0.05)]. Error bars indicate SD.

In the above experiments, we showed that *IDH* complements *DJ-1β* mutation in *Drosophila*. Using mouse DA cell line SN4741 [41] and CRISPR/Cas9-mediated genome editing, we generated *DJ-1* null DA cells to further investigate the relationship between *DJ-1β* and *IDH* in mammalian systems (S7A and S7B Fig). When we examined *IDH1* and *IDH2* mRNA expression in SN4741 cells under oxidative stress, there was no significant difference in *IDH1* mRNA expression between wild type and *DJ-1* null SN4741 cells with or without H_2_O_2_ treatment (Fig 4A). In contrast, *IDH2* mRNA expression in wild type SN4741 was elevated with H_2_O_2_ treatment, but it was almost nullified in *DJ-1* null SN4741 cells (Fig 4B). Moreover, *Keap1* knockdown completely rescued *IDH2* mRNA expression in H_2_O_2_-treated *DJ-1* null cells, showing that Nrf2 also mediates between mitochondrial IDH and DJ-1 in mammalian cells (Fig 4B and S7C Fig).

In MTT assays, cell viability increased when *IDH1* and *IDH2* were expressed in *DJ-1* null SN4741 cells under oxidative stress (Fig 4C). We performed both annexin V and propidium iodide (PI) staining to observe cell death, but only PI staining was positive in *DJ-1* null SN4741 cells in H_2_O_2_-containing media, indicating necrotic cell death (Fig 4D). Overexpression of *IDH1* or *IDH2* rescued this necrotic cell death upon H_2_O_2_ treatment in *DJ-1* null SN4741 cells (Fig 4D and 4E). These results in mammalian DA cells were highly similar to the *DJ-1* mutant phenotype recovery by IDH overexpression in *Drosophila* except that IDHc overexpression failed to protect DA neurons in some DA neuron clusters of *DJ-1β* mutants (Fig 2 and S4 Fig). Moreover, CM-H_2_DCFDA showed that intracellular ROS level dramatically increased in *DJ-1* null SN4741 cells when H_2_O_2_ was treated, but *IDH1* and *IDH2* overexpression significantly decreased the ROS level (Fig 4F and 4G). Furthermore, when mitochondrial ROS level was measured by MitoSOX, mitochondrial ROS drastically increased by oxidative stress in *DJ-1* null SN4741. However, the mitochondrial ROS level also decreased with overexpression of IDH1 and IDH2 in *DJ-1* null SN4741 (Fig 4H and 4I). These results suggested that the relationship between *DJ-1* and *IDH* genes is evolutionarily well conserved and is important in controlling intracellular ROS level, especially mitochondrial ROS level. In addition, a mitochondrial-specific ROS scavenger, MitoTEMPO, successfully inhibited oxidative stress-induced cell death in *DJ-1* null SN4741 cells (Fig 4J), further supporting the roles of DJ-1 in controlling mitochondrial ROS.

### Trimethyl isocitrate (TIC) protects *DJ-1* null DA cells from oxidative stress

**Fig 5:**
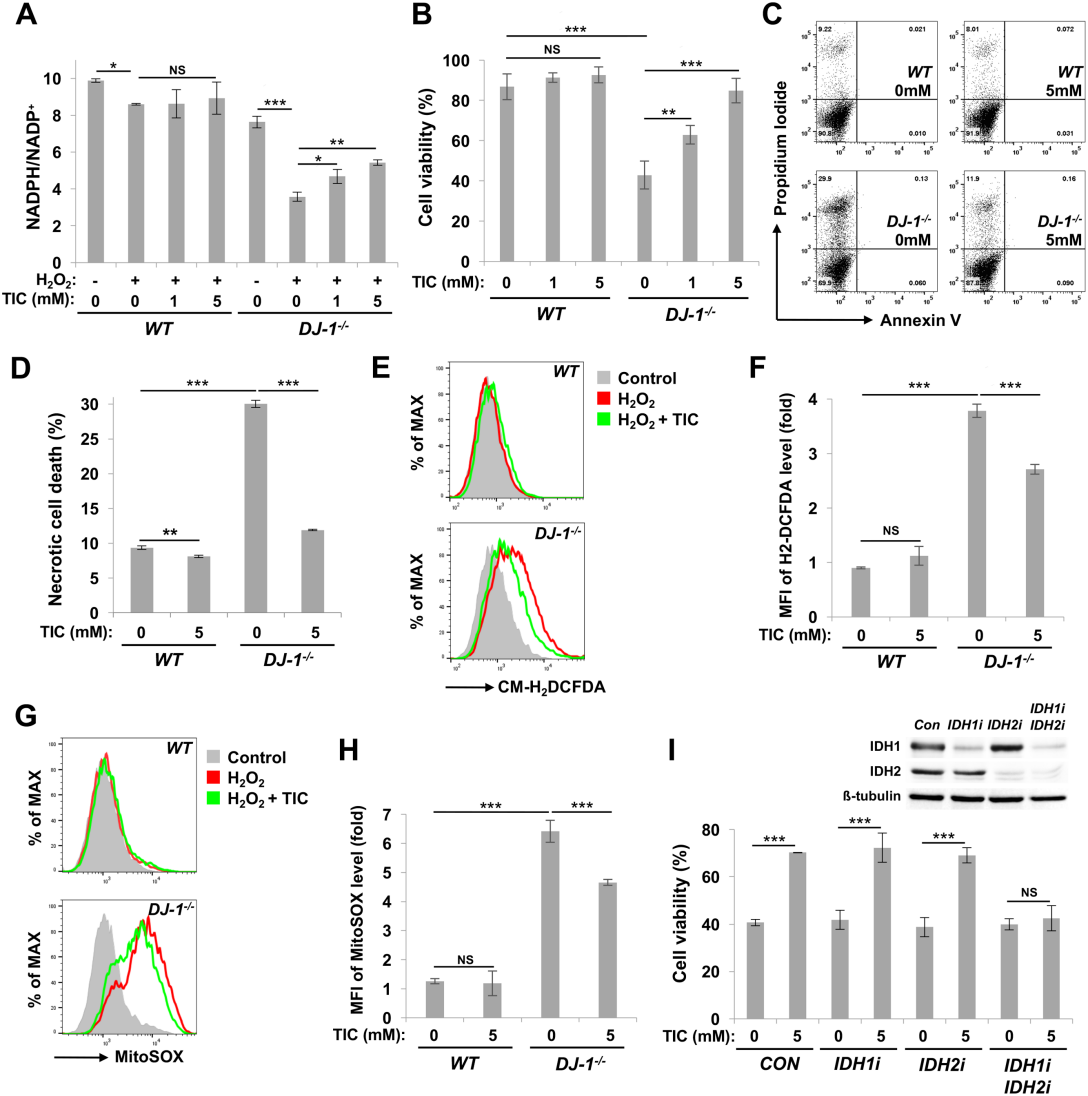
TIC protects mammalian DA cells against oxidative stress. (A) NADPH/NADP^+^ ratio measured in H_2_O_2_-treated wild type (*WT*) and *DJ-1* null (*DJ-1*^*-/-*^) SN4741 cells with pre-treatment of TIC at indicated concentrations (n = 3). (B) Cell viability of H_2_O_2_-treated SN4741 cells with pre-treatment of TIC at indicated concentrations. Cell viability was measured by MTT assays (n = 3). (C) Propidium iodide and annexin V FITC staining of H_2_O_2_-treated SN4741 cells. TIC was pre-treated with indicated concentrations. (D) Necrotic cell death rates (n = 3). (E) Flow cytometric analysis of CM-H_2_DCFDA-stained SN4741 cells. Gray-filled area: *WT* or *DJ-1* null SN4741 cells without H_2_O_2_ treatment; Red line: H_2_O_2_-treated SN4741 cells; Green line: TIC and H_2_O_2_-treated SN4741 cells. TIC was pre-treated with indicated concentrations. (F) Fold change in the mean of fluorescence intensity (MFI) of CM-H_2_DCFDA in flow cytometric analysis (n = 3). (G) Flow cytometric analysis of MitoSOX-stained SN4741 cells. Gray-filled area: *WT* or *DJ-1* null SN4741 cells without H_2_O_2_ treatment; Red line: H_2_O_2_-treated SN4741 cells; Green line: TIC and H_2_O_2_-treated SN4741 cells. TIC was pre-treated with indicated concentrations. (H) Fold change in MFI of MitoSOX in flow cytometric analysis (n = 3). (I) Cell viability of H_2_O_2_-treated *DJ-1* null SN4741cells transfected with control (*CON*) siRNA, *IDH1*-specific siRNA (*IDH1i*) or *IDH2*-specific siRNA (*IDH2i*). To downregulate gene expression of both *IDH*s, *IDH1*- and *IDH2*-specific siRNA were co-transfected (*IDH1i IDH2i*). TIC was pre-treated with indicated concentrations. Cell viability was measured by MTT assays (n = 3). The inset shows immunoblot of endogenous IDH1 and IDH2. β-tubulin as a loading control. Data information: Significance was determined by one-way ANOVA with Sidak correction (*, P<0.05; **, P<0.01; ***, P<0.001; NS, not significant). Error bars indicate SD.

To further investigate the antioxidant effect of IDH, we searched for a compound that increases IDH activity, but found no such existing activator. To increase NADPH production by IDH, we tried to deliver isocitrate, a substrate of IDH, into the cells. We decided to attach 3 methyl groups to isocitrate to make it permeable to the cells. When treated with H_2_O_2_, NADPH/NADP^+^ ratio in *DJ-1* null SN4741 cells decreased dramatically compared to wild type cells (Fig 5A). This result showed that DJ-1 is indispensable for maintaining NADPH/NADP^+^ ratio under oxidative stress. Surprisingly, trimethyl isocitrate (TIC) treatment substantially restored NADPH/NADP^+^ ratio (~40–50%) and cell viability (~70–80%) dose-dependently in *DJ-1* null SN4741 cells under oxidative stress (Fig 5A and 5B), suggesting that TIC successfully crossed through the plasma membrane and increased NADPH production under oxidative stress. In an additional experiment using annexin V and PI, PI uptake was decreased when cells were pre-treated with TIC (Fig 5C and 5D). These results indicate that TIC inhibited necrotic cell death caused by H_2_O_2_ treatment. When SN4741 cells were stained with ROS indicators, CM-H_2_DCFDA and MitoSOX, intracellular and mitochondrial ROS levels were increased in H_2_O_2_-treated *DJ-1* null SN4741 cells. However, when cells were pre-treated with TIC, ROS levels immensely decreased in H_2_O_2_-treated *DJ-1* null SN4741 cells (Fig 5E–5H). These results implied that TIC raises NADPH/NADP^+^ ratio which detoxifies ROS and ultimately protects *DJ-1* null DA cells from oxidative stress. To see whether IDH is indeed responsible for the cell protecting effect of TIC, we suppressed *IDH* expression in *DJ-1* null SN4741 cells with siRNA technology. We observed that when gene expression of *IDH1* and *IDH2* was blocked simultaneously, TIC had no effect in protecting cells against oxidative stress, confirming that TIC functions through IDH (Fig 5I).

## Discussion

Recently, it has been reported that oxidative stress and mitochondrial maintenance are highly correlated with PD [2]. In this study, we performed RNA-seq to find a novel gene which provides resistance against oxidative stress at the downstream of DJ-1. We assumed that there are important target genes related to the mitochondrion, which is the main organelle of ROS generation. Through RNA-seq analysis (Fig 1A and S1A and S1B Fig), we found that *IDH* was a gene included in mitochondrion ontology downstream of DJ-1. Loss-of-function flies for *IDH* were generated, whose *in vivo* ROS levels were increased (Fig 1G–1H). These *IDH* mutants displayed reduced survival rates against oxidative stress and decreased DA neurons and climbing ability (Fig 1I–1K, and S3E and S3F Fig). *IDH* mutant flies showed decreased ATP generation and mtDNA contents (S3A–S3D Fig) that were also observed in *IDH2* knockout mouse [42]. These phenotypes are very similar with the PD-related phenotypes of *PINK1*, *Parkin* and *DJ-1β* mutant flies [18, 27, 32, 43, 44], supporting that *IDH* is highly implicated in PD pathology. Consistently, under MPTP treatments, *IDH2* KO mouse showed more severe PD-related phenotypes than those of wild type controls [45]. More interestingly, IDH overexpression in *DJ-1β* null mutants rescued the PD-related phenotypes of *DJ-1β* mutants (Fig 2), but not those of *PINK1* null flies (S6 Fig). This specific rescue of the phenotypes in *DJ-1β* mutants implies that IDH is a unique downstream regulator of *DJ-1*-dependent and/or oxidative stress-induced PD pathologies.

In searching for the molecular link between DJ-1 and IDH, overexpression of Nrf2, a well-known transcription factor that responds to oxidative stress [33], suppressed the loss of DA neurons in *DJ-1β* mutants under oxidative stress (Fig 3H–3K). Further genetic analysis demonstrated that *Keap1* loss-of-function mutation restores decreased *IDH* expression and survival rates of *DJ-1β* mutants under oxidative stresses (Fig 3A–3E), supporting Nrf2 as the molecular mediator that links DJ-1 and IDH under oxidative stress. Interestingly, only the expression of mitochondrial IDH isoforms was suppressed by *DJ-1* mutation in *Drosophila* and mouse SN4741 DA cells under oxidative stress (Figs 2E–2G, 4A and 4B). This specific regulation of mitochondrial *IDH*s is mediated by the Keap1-Nrf2 pathway in *Drosophila* (Fig 3A–3C, 3F and 3G) and mammalian system (Fig 4B). When these mitochondrial IDHs were overexpressed, oxidative stress-induced *DJ-1* null defects, including DA neuron loss in *Drosophila* brain, decreased *Drosophila* survival rates, and increased intracellular and mitochondrial ROS levels in SN4741 DA cells, were successfully rescued (Figs 2 and 4). These data established the role of the DJ-1-Nrf2-IDH pathway in DA neuronal protection against oxidative stress and implied that DJ-1 effectively eliminates intracellular ROS by reducing ROS generation in mitochondria through mitochondrial IDHs.

Although most data converge on this conclusion, there are some results that may raise further questions. In *Drosophila* and SN4741 cells, cytosolic IDHs can also ameliorate the *DJ-1* null defects mentioned above, including increased mitochondrial ROS levels. Is regulating mitochondrial ROS levels indeed important to protect cells? Shin et al. reported that reducing cytosolic ROS level decreases mitochondrial ROS levels [46]. Consistently, in our experiment, overexpression of IDH1, a cytosolic mammalian IDH, substantially reduced mitochondrial ROS accompanying decreased cellular ROS in SN4741 cells (Fig 4I). However, IDH2, the mitochondrial counterpart, reduced more mitochondrial ROS than IDH1 in SN4741 cells (Fig 4I). In *Drosophila* brains, IDHc failed to protect DA neurons in some DA neuron clusters (Fig 2C, 2D and S4 Fig). These results indicated that indirect elimination of mitochondrial ROS by cytosolic IDHs is not sufficient to protect cells compared to direct elimination via mitochondrial IDHs, especially in DA neurons that may have more complex and varied stresses compared to cultured cells. Furthermore, mitochondria-specific antioxidant MitoTEMPO strongly inhibited cell death induced by oxidative stress in *DJ-1* null SN4741 cells, further confirming the importance of mitochondrial ROS reduction in DJ-1-mediated anti-oxidative stress responses (Fig 4J). In addition, although all *Drosophila IDH*s are encoded from the same gene locus, only the expression of mitochondrial isoforms is regulated by DJ-1 and CncC (Fig 3). In DNA sequence analyses, we found a putative CpG island between the transcription start sites of IDHc and IDHm1 and 2, raising the possibility that DNA methylation inhibits CncC to induce IDHc expression (S2C Fig). However, we could not find the experimental evidence that DNA methylation is involved in the mitochondrial *IDH*-specific expression, so the molecular mechanism of this expression will be a future topic.

**Fig 6:**
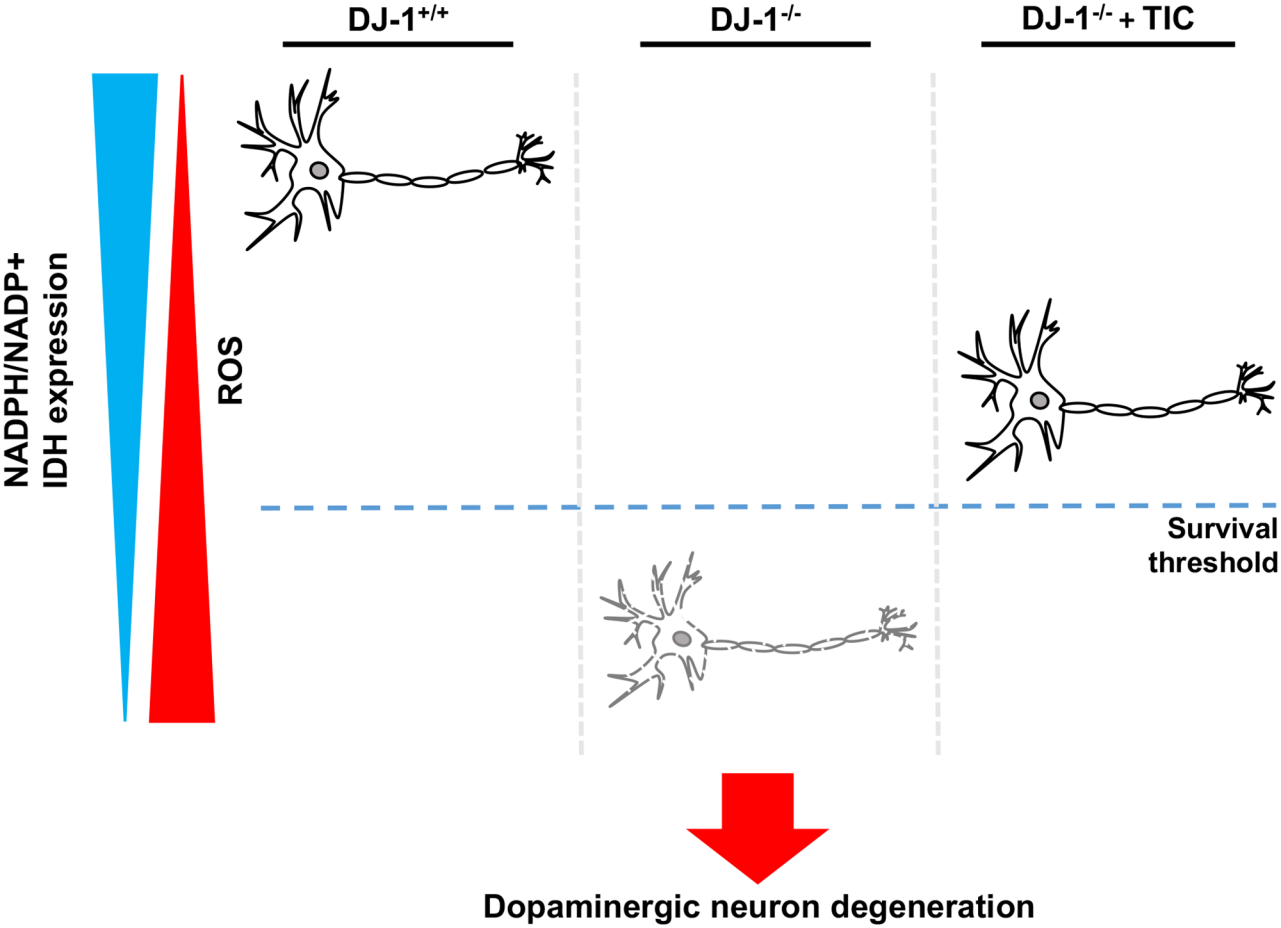
Schematic models of the protective effects of IDH on DA neurons against oxidative stress in *DJ-1* null background.

After confirming IDH as a downstream target of DJ-1, we investigated recent issues on IDH research based on our findings. It has been reported that *IDH1* and *IDH2* neomorphic mutations are prevalent in various cancers [29]. These mutant IDH enzymes convert α-ketoglutarate into D-2-hydroxyglutarate, which promotes tumorigenesis [47]. Therefore, we tested whether these neoenzymes can affect *DJ-1* mutant phenotypes. Overexpression of *Drosophila* IDHm1 R166K and R134Q mutants, which correspond to the cancer-associated human IDH2 R172K and R140Q mutants, respectively [29], failed to increase IDH activity in flies and restore the decreased survival rates and DA neuron numbers of *DJ-1β* mutants (S8 Fig). This is consistent with the reports that the cancer-associated IDH mutants do not produce, but consume NADPH [47]. In contrast, wild type IDHm1 consistently increased *in vivo* IDH activity and rescued the PD-related phenotypes (S8 Fig). Thus, these results implicate that the activity of wild type IDH, not the cancer-related one, is linked to *DJ-1*-associated PD, suggesting that the drugs developed to target cancer-related IDH mutant enzymes are not appropriate to treat *DJ-1*-associated PD. To overcome this limitation, we hypothesized that excess concentration of cell-permeable isocitrate, the substrate of IDH, would help treat *DJ-1*-associated PD by raising NADPH/NADP^+^ ratio to increase the reducing power in the cell. As expected, TIC substantially elevated NAPDH/NADP^+^ ratio and strongly reduced intracellular and mitochondrial ROS levels in H_2_O_2_-treated SN4741 cells (Fig 5A and 5E–5H). TIC also increased survival rate and lowered necrotic cell death against oxidative stress (Fig 5B–5D). *IDH* expression knockdown inhibited this TIC-mediated cell protection, supporting the idea that TIC protects cells via IDH (Fig 5I). Since TIC treatment significantly increased cell viability in *DJ-1* null SN4741 cells, we expected a similar degree of increase in NADPH/NADP^+^ ratio. However, the observed ratio was less than expected, indicating that NADPH is being rapidly used to resist oxidative stress (Fig 5A). Overall, these results confirmed the protective role of IDH against oxidative stress, and also suggested cell-permeable isocitrates as putative drug candidates for the treatment of *DJ-1* deficiency-associated human pathology including PD (Fig 6).

## Materials and methods

### *Drosophila* strains

*da*-GAL4, *hs*-GAL4, and *elav*-GAL4 strains were obtained from the Bloomington Stock Center. *IDH*^*P*^ mutants (G9298) were obtained from KAIST-GenExel *Drosophila* library and backcrossed to *w*^*1118*^ controls for 6 generations to remove genetic background effects. The insertion site of the P-element in *IDH*^*P*^ is located at +301 of *IDHm1* ORF, +244 of *IDHm2* ORF, and +205 of *IDHc* ORF. A revertant (*IDH*^*RV*^) was generated by precise excision of the P-element in *IDH*^*P*^ after backcrossing. In DNA sequencing analysis, *IDH*^*RV*^ showed a precise excision of the P-element with no insertion or deletion of nucleotides. IDHm1, IDHm2, IDHc, and CG17352 cDNAs were sub-cloned from GH01524, RE70927, AT04910 and GH02239 BDGP cDNA clones, respectively. IDHm1 R134Q and R166K mutant cDNAs were generated by QuikChange^™^ site-directed mutagenesis kit (Agilent Technologies) using following primer pairs: IDHm1 R134Q F (gcc caa cgg tac cat cca aaa cat ctt ggg agg aac), R134Q R (gtt cct ccc aag atg ttt tgg atg gta ccg ttg ggc), R166K F (gaa gcc tat tgt gat cgg taa aca tgc cca cgc cga tca gt) and R166K R (act gat cgg cgt ggg cat gtt tac cga tca caa tag gct tc). The IDH cDNAs were inserted into the pUAST vector with C-terminal HA-tag and microinjected into *w*^*1118*^ embryos. The CG17352 cDNA was inserted into the pACU2 vector and microinjected into *y*^*1*^ *w*^*1118*^; *PBac{y+-attP-3B}VK00001* embryos. *PINK1*^*B9*^, *DJ-1β*^*ex54*^ and *UAS-DJ-1β* flies were generated as previously described [18, 32]. The *tyrosine hydroxylase* (*TH*)-GAL4 fly was a gift from Dr. S. Birman. The *Keap1*^*EY5*^, *UAS-Keap1* and *UAS-CncC* lines were provided by Dr. D. Bohmann.

### Oxidative stress assays and life span assays

For oxidative stress assay, three or four groups of 3-day-old 30 male flies (n = 90 or 120) were starved for 6 h and transferred to a vial containing a gel of phosphate-buffered saline (PBS), 5% sucrose and an oxidative stress agent (5 mM rotenone or 1% H_2_O_2_) as indicated in figure legends. Dead flies were counted at the indicated time points. For life span assay, three or four groups of 30 male flies (n = 90 or 120) were transferred to fresh fly food vials and scored for survival every 3 or 4 days.

### Climbing assays

To check climbing activity of *IDH*^*P*^ mutants, groups of fifteen 3- or 30-day-old males grown on normal media were transferred into climbing ability test vials and incubated for 1 h at room temperature for environmental acclimatization. After tapping the flies down to the bottom, the number of climbing flies was counted for 10 seconds. For each group, ten trials were performed, and the climbing score (percentage ratio of the number of climbed flies against the total number) was obtained. To check climbing ability of IDH expressing *DJ-1β* mutant males under oxidative stress, groups of 3-day-old 30 males were starved for 6 h and transferred to a vial containing a gel of phosphate-buffered saline (PBS), 5% sucrose and 0.5 mM rotenone. After 4 days, they were re-grouped in size of fifteen and tested according to the procedures above. The average climbing score with standard deviation was calculated for five independent tests.

### mtDNA PCR and ATP assay

For mtDNA PCR, total DNA from five thoraces of 3- or 30-day-old male flies was extracted. Then, quantitative real-time PCR was performed as previously described [32]. Genomic DNA levels of *rp49* were measured for internal controls. The results were expressed as fold changes relative to the control. For ATP assay, five thoraces from 3-day-old male flies were dissected, and ATP concentration was measured as previously described [32]. The relative ATP level was calculated by dividing the measured ATP concentration by the total protein concentration. Protein concentration was determined by a bicinchoninic acid (BCA) assay (Sigma). In the mtDNA PCR and ATP assay, the average value with standard deviation was obtained from three independent experiments.

### S2 cell culture, transfection, and immunocytochemistry

S2 cells were cultured and transiently transfected with IDHm1, IDHm2, and IDHc plasmids used to generate UAS-IDH flies as described previously [48]. To induce IDH protein expression in pUAST vector, we co-transfected pMT-GAL4 plasmids that contained GAL4 gene with metallothionein promoter. Twenty-four hours before cell staining, CuSO4 was treated to induce expression of GAL4 and IDHs. Cells were pre-incubated with 5 μg/mL MitoTracker Red CMXRos (Molecular Probes) for 1 h at 25°C and then subjected to the standard immunocytochemistry using anti-HA antibody (Invitrogen).

### *Drosophila* DA neuron and tissue staining

To check the change of the DA neuron numbers in *DJ-1β* mutants under oxidative stress, 30 male flies (3-day-old) were starved for 6 h and incubated for 3 days in a vial containing a gel of phosphate-buffered saline (PBS), 5% sucrose and an oxidative stress agent (0.2 mM rotenone or 1% H_2_O_2_). To check the change of the DA neuron numbers in *IDH* or *PINK1* mutants without oxidative insults, 30 male flies were transferred to a fresh normal media vial every 3 or 4 days for the time points indicated in figure legends. To stain DA neurons, adult brains from ten randomly chosen flies were fixed with 4% paraformaldehyde and stained with anti-TH rabbit antibody (1:50, Pel-Freez, P40101-150) as previously described [32]. Brains were observed and imaged by LSM 700 confocal microscope (Zeiss). For imaging ROS production in fly tissues, the indirect flight muscles from 3-day-old males were dissected in Schneider’s medium (Sigma) and incubated for 5 min in Schneider’s medium containing 30 μM dihydroethidium (DHE, Invitrogen). Muscles were observed and imaged by BX-50 microscope (Olympus).

### Genotypes

IDH^RV^ (*IDH*^*RV*^/*IDH*^*RV*^); *IDH*^*P*^ (*IDH*^*P*^/*IDH*^*P*^); *hs* (*hs-GAL4*/*+*); *hs>CG17352* (*hs-GAL4/UAS-CG17352*); *hs DJ-1β*^*ex54*^ (*hs-GAL4/+*; *DJ-1β*^*ex54*^*/DJ-1β*^*ex54*^); *hs>IDHm1 DJ-1β*^*ex54*^ (*hs-GAL4/UAS-IDHm1*; *DJ-1β*^*ex54*^*/DJ-1β*^*ex54*^); *hs>IDHm2 DJ-1β*^*ex54*^ (*hs-GAL4/UAS-IDHm2*; *DJ-1β*^*ex54*^*/DJ-1β*^*ex54*^); *hs>IDHc DJ-1β*^*ex54*^ (*hs-GAL4/UAS-IDHc*; *DJ-1β*^*ex54*^*/DJ-1β*^*ex54*^); *elav* (*elav-GAL4*/*+*); *elav DJ-1β*^*ex54*^ (*elav-GAL4/+; DJ-1β*^*ex54*^*/DJ-1β*^*ex54*^); *elav>IDHm1 DJ-1β*^*ex54*^ (*elav-GAL4/UAS-IDHm1; DJ-1β*^*ex54*^*/DJ-1β*^*ex54*^); *elav>IDHm2 DJ-1β*^*ex54*^ (*elav-GAL4/UAS-IDHm2; DJ-1β*^*ex54*^*/DJ-1β*^*ex54*^); *elav>IDHc DJ-1β*^*ex54*^ (*elav-GAL4/UAS-IDHc*; *DJ-1β*^*ex54*^*/DJ-1β*^*ex54*^); *DJ-1β*^*ex54*^ (*DJ-1β*^*ex54*^*/DJ-1β*^*ex54*^); *Keap1*^*EY5/+*^ (*Keap1*^*EY5*^*/+*); *DJ-1β*^*ex54*^ *Keap1*^*EY5/+*^ (*DJ-1β*^*ex54*^*Keap1*^*EY5*^*/DJ-1β*^*ex54*^); *WT* (*+*/*Y*); *elav>CncC DJ-1β*^*ex54*^ (*elav-GAL4/UAS-CncC; DJ-1β*^*ex54*^*/DJ-1β*^*ex54*^); *hs>CncC* (*hs-GAL4 UAS-CncC/+*); *hs>CncC Keap1* (*hs-GAL4 UAS-CncC/UAS-Keap1*); *hs>CncC Keap1 DJ-1β* (*hs-GAL4 UAS-CncC/UAS-Keap1*; *UAS-DJ-1β/+*); *IDH*^*P*^ *DJ-1β*^*ex54*^ (*IDH*^*P*^/*IDH*^*P*^*; DJ-1β*^*ex54*^*/DJ-1β*^*ex54*^); *da* (*da-GAL4*/*+*); *B9 da* (*PINK1*^*B9*^/Y;; *da-GAL4*/*+*); *B9 da>IDHm1* (*PINK1*^*B9*^/Y; *UAS-IDHm1/+*; *da-GAL4*/+); *B9 da>IDHc* (*PINK1*^*B9*^/Y; *UAS-IDHc/+*; *da-GAL4*/+); *TH* (*TH-GAL4*/*+*); *B9 TH* (*PINK1*^*B9*^/Y;; *TH-GAL4*/*+*); *B9 TH>IDHm1* (*PINK1*^*B9*^/Y; *UAS-IDHm1/+*; *TH-GAL4*/+); *B9 TH>IDHc* (*PINK1*^*B9*^/Y; *UAS-IDHc/+*; *TH-GAL4*/+); *hs>IDHm1* (*hs-GAL4/UAS-IDHm1*); *hs>IDHm1*^*RQ*^ (*hs-GAL4/UAS-IDHm1*^*R134Q*^); *hs>IDHm1*^*RK*^ (*hs-GAL4/UAS-IDHm1*^*R166K*^); *hs>IDHm1*^*RQ*^ *DJ-1β*^*ex54*^ (*hs-GAL4/UAS-IDHm1*^*R134Q*^; *DJ-1β*^*ex54*^*/DJ-1β*^*ex54*^); *hs>IDHm1*^*RK*^ *DJ-1β*^*ex54*^ (*hs-GAL4/UAS-IDHm1*^*R166K*^; *DJ-1β*^*ex54*^*/DJ-1β*^*ex54*^); *elav>IDHm1*^*RQ*^ *DJ-1β*^*ex54*^ (*elav-GAL4/UAS-IDHm1*^*R134Q*^; *DJ-1β*^*ex54*^*/DJ-1β*^*ex54*^); *elav>IDHm1*^*RK*^ *DJ-1β*^*ex54*^ (*elav-GAL4/UAS-IDHm1*^*R166K*^*; DJ-1β*^*ex54*^*/DJ-1β*^*ex54*^).

### Luciferase assay

To measure transactivation activity of CncC on the IDH gene, the promoter and 5’ untranslated region (S1C Fig) were subcloned into pGL3 reporter plasmid (Promega) using following primers: IDH promoter F (gcg ggt acc cag tta ttc gct gcg tct gat tgg) and IDH promoter R (gcg gga tcc gaa ccg acc gac gac tgg aaa cg). For generating the IDH reporter with ARE mutation, the first five bases (TGACG) of the putative ARE (TGACGGGGC) were deleted by QuikChange^™^ site directed mutagenesis kit (Agilent Technologies). S2 cells were transfected with wild type or ARE mutant IDH reporter, pUAST-CncC, pRL-TK Renilla reporter, and pMT-GAL4 plasmids. Two days later, CncC expression was induced by CuSO4 treatment. After 24 h, luciferase assays were performed using Dual-Luciferase^™^ reporter assay kit (Promega) according to the manufacturer's instructions. The average luciferase activity with standard deviation was obtained from three independent experiments.

### Quantitative RT-PCR

Total RNA from heads, thoraces, abdomens, or whole bodies of 3-day-old flies or SN4741 cells was extracted and reversely transcribed as previously described [49]. To check the inhibition of *IDH* expression in *IDH*^*P*^ mutants, 5 whole bodies were used (Fig 1D and S2D Fig). To check the expression change of *IDH* and its isoforms in *DJ-1β* mutants, 5 heads and, thoraces, reported to be predominantly damaged in PD-gene-defected flies were used (Figs 1D and 2E–2G) [32]. To check whether the expression change of *IDH* is tissue-specific, 10 heads, 10 thoraces, or 10 abdomens were used (S1C–S1E Fig). To confirm the gene expression of each isoform, 5 whole bodies were used (Fig 3A–3C, 3F and 3G). SN4741 cells were seeded in 6-well plates at a density of 1 × 10^6^ cells per well. Then, quantitative real-time PCR was performed using SYBR Premix Ex Taq (Takara) on Prism 7000 Real-Time PCR System (ABI). *rp49* levels or mouse actin levels were measured for internal control of *Drosophila* or SN4741 samples, respectively. The results were expressed as fold changes relative to the control. The average mRNA level with standard deviation was obtained from three independent experiments. For primer pairs, we used rp49-F (gct tca aga tga cca tcc gcc c) and rp49-R (ggt gcg ctt gtt cga tcc gta ac), IDH-F (cct tcc tgg aca ttg agc tg) and IDH-R (gta ccg ttg ggc gac ttc cac), CG17352-F (cac atc tcg ttg aga gtg gat gac) and CG17352-R (cga atg tag tag cca ttg agg atg), hsp22-F (gtc ctg acc atc agt gtg c) and hsp22-R (cca gtc tgc tcg atg gtc ac), IDHm1-F (cat cag cgc cgc gat gg) and IDH-R (gta ccg ttg ggc gac ttc cac), IDHm2-F (gtg agc gag atg gcc cag aag) and IDH-R (gta ccg ttg ggc gac ttc cac), IDHc-F (gta tgc tct ccc gaa cag atg g) and IDH-R (gta ccg ttg ggc gac ttc cac), mouse IDH1-F (cct ggg cct gga aaa gta ga) and mouse IDH1-R (tcc tgg ttg tac atg ccc at), mouse IDH2-F (cta tga cgg gcg ttt caa gg) and IDH2-R (cct tga gcc agg atg tca ga), mouse actin-F (ttc ttt gca gct cct tcg tt) and mouse actin-R (tgg atg gct acg tac atg gc), and mouse Keap1-F (tgc ccc tgt ggt caa agt g) and mouse Keap1-R (ggt tcg gtt acc gtc ctg c).

### Mammalian cell culture and transfection

SN4741 cells were established from the substantia nigra region of wild type and *DJ-1* knock out mouse embryos, and were characterized for expression of the neuronal markers including TuJ1 and NeuN, and the DA cell marker TH as previously described [41]. The SN4741 cells were grown in RF medium (DMEM supplemented with 10% fetal bovine serum, 1% glucose, and 2 mM L-glutamine) at 33°C in a humidified atmosphere with 5% CO_2_. pCMV14 vector, pCMV14 FLAG-IDH1, or pCMV14-IDH2 was transfected using Lipofectamine Plus Reagent (Invitrogen) according to the manufacture’s protocol. siRNAs for control (Bioneer, #SN-1003), mouse IDH1 (Bioneer, #1371568), mouse IDH2 (Bioneer, #1371576), or mouse Keap1 (Bioneer, #1367293) was transfected to SN4741 cells using the RNAiMAX reagent (Invitrogen) according to the manufacture’s protocol.

### Generation of *DJ-1* null SN4741 knockout cell line

CRISPR genome editing technique was used for the deletion of *DJ-1*. The guide RNA sequence (gtg gat gtc atg cgg cga gc) was cloned into the px459 vector. The plasmid was transfected into SN4741 cells. 48 h after transfection, transfected cells were selected by 5 μg/mL puromycin for 3 days and then single colony was transferred onto 96-well plates with one colony in each well. The clones were screened by immunoblot with anti-DJ-1 antibody (1:1,000, Novus Biology, #NB100-483).

### Immunoblot

For detection of IDH1, IDH2, β-tubulin, and HA- or FLAG-tagged protein, S2 or SN4741 cells were lysed with Lysis Buffer [48]. The lysates were purified by centrifugation and boiled in SDS sample buffer. The samples were subjected to SDS-PAGE and proteins were transferred to nitrocellulose membrane. The membrane was incubated for 30 min in Blocking Solution and further incubated with anti-IDH1 antibody (1:1,000, Bethyl, #A304-162A-T), anti-IDH2 antibody (1:1,000, Bethyl, #A304-096A-T), anti-FLAG antibody (1:1,000, MBL, #M185-3L), anti-HA antibody (1:1,000, Invitrogen, #26183), anti-DJ-1 antibody (1:1,000, Novus Biology, #NB100-483), or anti-β-tubulin antibody (1:1,000, DSHB, Clone E7) as described previously [48]. Membrane-bound antibodies were detected with ImageQuant LAS 4000 system (GE Healthcare Life Sciences).

### MTT (3-[4,5-dimethylthiazol-2-yl]-2,5-diphenyltetrazolium bromide) assay

Cells were seeded in 12-well plates at a density of 6 × 10^5^ cells per well. After pre-treatment of TIC (LegoChem Biosciences) or MitoTEMPO (Sigma, 10 nM) at the indicated concentrations for 1 h, cells were treated with 1.5 mM H_2_O_2_. After 6 h incubation, the culture medium was removed and replaced with a medium containing 0.5 mg/mL of MTT dissolved in PBS (pH 7.2). After 4 h, the formed formazan crystals were dissolved in 400 μL of DMSO, and the absorbance intensity was measured at a wavelength of 595 nm using Infinite 200 pro (TECAN). The relative cell viability was expressed as a percentage relative to the untreated control cells. The average viability with standard deviation was obtained from three independent experiments.

### Annexin V staining

SN4741 cells were seeded in 6 well plates with cell density of 1 × 10^6^ cells per well. Treatment of TIC (5 mM) and H_2_O_2_ (1.5 mM) was performed as described above. The cells were stained using the Annexin V-FITC Apoptosis Detection kit (BD Biosciences) according to the manufacturer's protocol. Stained cells were analyzed by flow cytometry using BD FACSCanto II (BD sciences). A total of 10,000 events was analyzed for each sample, and the necrotic cell death rates obtained from three independent experiments were presented as the mean values with standard deviations.

### Measurement of intracellular ROS levels

SN4741 cells were pre-treated with TIC (5 mM) for 1 h. Following 2 h treatment of 1.5 mM H_2_O_2_, cells were incubated with 5 μM of 5- and 6-chloromethyl-2′,7′-dichlorodihydrofluorescein diacetate (CM-H_2_DCFDA, Invitrogen) for 30 min at 33°C. The cells were trypsinized, washed with PBS, suspended in PBS, and analyzed with BD FACSCanto II (BD sciences). A total of more than 5,000 events was analyzed for each sample, and the results obtained from three independent experiments were presented as the mean values with standard deviations.

### Measurement of mitochondrial ROS levels

SN4741 cells were pre-treated with TIC (5 mM) for 1 h. Following 2 h treatment of 1.5 mM H_2_O_2_, cells were incubated with 1 μM MitoSOX (Invitrogen) for 10 min at 33°C. The cells were trypsinized, washed with PBS, suspended in PBS, and analyzed with BD FACSCanto II (BD sciences). A total of more than 5,000 events was analyzed for each sample, and the results obtained from three independent experiments were presented as the mean values with standard deviations.

### Measurement of NADPH/NADP^+^ ratio

SN4741 cells were pre-treated with TIC (5 mM) for 1 h. Following 6 h treatment of 1.5 mM H_2_O_2_, the cells were lysed with 0.2 N NaOH with 1% dodecyl trimethyl ammonium bromide (DTAB, Sigma). To measure NADPH/NADP^+^ ratio in flies, five 3-day-old male flies were homogenized in 0.2 N NaOH with 1% DTAB. Samples were centrifuged to obtain supernatants. NADP^+^ and NADPH levels of the lysates were individually measured by using NADP/NADPH-glo^™^ assay kit (Promega) according to the manufacturer's instructions, and NADPH/NADP^+^ ratio was calculated. The average NADPH/NADP^+^ ratio with standard deviation was obtained from three independent experiments.

### Measurement of IDH activity

Ten 3-day-old male flies were homogenized in 40 mM Tris buffer (pH 7.4). Supernatants from samples were each added to the Tris buffer-containing NADP^+^ (2 mM), MgCl_2_ (2 mM), and isocitrate (5 mM). IDH activity was determined by monitoring the kinetics of NADPH production at 340 nm at 25°C with SpectraMax M2 multi-mode microplate reader (Molecular Devices). The average relative IDH activity with standard deviation was obtained from three independent experiments.

### Statistical analysis

For quantification of DA neurons, four major DA neuron clusters from more than 15 brains of each genotype were observed in a blind fashion to eliminate bias (n = 30~40). To compare three or more groups, we used one-way ANOVA with Sidak correction. For two-group comparison, we used Student’s two-tailed t test. The Kaplan-Meier estimator and the log-rank test were conducted on the survival data to determine whether each treatment had any effect on the longevity of individuals using Online Application Survival Analysis Lifespan Assays (http://sbi.postech.ac.kr/oasis). All n values defined in the figure legends refer to biological replicates unless otherwise indicated. The experiments were not randomized. To obtain consistent results, we incubated flies for at least three days after eclosion and excluded dead or malformed flies before any fly assay in this report.

### RNA-sequencing data analysis

20 male flies (3-day-old) were starved for 6 h and transferred to a vial containing a gel of PBS, 5% sucrose and 5 mM rotenone. 16 h later, total RNA from ten heads and thoraces of ten randomly chosen stressed flies was extracted. Indexed RNA-seq libraries were constructed using Illumina TruSeq RNA Sample Prep Kit version 2. Each library was sequenced in paired end using Illumina HiSeq2500 platform. Raw reads (n = 3) were aligned to the Ensembl *Drosophila melanogaster* reference genome (BDGP6) using Tophat2. The read alignments were assembled into transcriptome assembly. Fragments per kilobase of transcripts per million reads (FPKM) as normalized expression levels were calculated using Cufflinks. The assemblies for each replicate were merged together using Cuffmerge. Differentially expressed gene (DEG) analysis was performed using Cuffdiff workflow to screen DEGs with false discovery rate (FDR) adjusted by *P*-value of < 0.05 and fold change of > 1.5. Gene ontology (GO) analysis was performed for term enrichment using g:Profiler and Amigo2. We filtered GO tree hierarchy and statistical significance threshold was FDR < 0.05. A volcano plot and hierarchical clustering in a heat map were generated by statistical package R.

## Supporting information

S1 Fig Gene expression analysis of mitochondrial redox proteins in *DJ-1* mutants under oxidative stress. (A) The Venn diagram summarizes the ontology analysis using the genes screened from RNA-seq of wild type and *DJ-1β* null flies under rotenone treatment. Red circle indicates the number of genes in oxidation-reduction process ontology, and green circle indicates the number of genes in mitochondrion ontology. 34 genes are the ones that fall into both ontologies, reduction-oxidation process and mitochondrion. (B) The heat map presents changes in the expression of the 34 genes mentioned in Fig. S1A. #1, #2, and #3 indicate each of three independent RNA-seq experiments (n = 3). (C-E) Comparison of *IDH* mRNA levels in heads (C), thoraces (D), and abdomens (E) of wild type flies (*WT*) and *DJ-1β* null mutants (*DJ-1β*^*ex54*^) under control (CON) or rotenone treatment (Rotenone) (n = 3).
(TIF)

S2 Fig Characterization of *Drosophila IDH* mutants. (A) Sequence alignment of *Drosophila* IDHs (IDHc, IDHm1, and IDHm2), human IDH1 (hIDH1), and human IDH2 (hIDH2). Mitochondrial targeting sequence, catalytic residues, and R134 and R166 residues were indicated. (B) Cytosolic and mitochondrial localization of IDH isoforms. Subcellular localization of C-terminally HA-tagged cytosolic IDH (IDHc) and mitochondrial IDHs (IDHm1 and IDHm2) in S2 cells was determined by co-staining with anti-HA antibody (green) and MitoTracker (red). Anti-HA immunoblots confirmed expression of each isoform. Scale bar: 5 μm. (C) Schematic genomic organization of the *IDH* locus. Black rectangles: coding sequences (CDS); gray rectangles: untranslated regions (UTR). Genomic structures of *IDH*^*P*^ were described in Materials and Methods. The location of the putative Antioxidant Response Element (ARE) (TGACGGGGC) and the promoter region in *IDH* reporter plasmids were also presented. Binding sites of Quantitative PCR primers for all IDH isoform genes (blue arrows) and each isoform (red arrows) were indicated. Sequences of the primers were described in Materials and Methods. A putative CpG island was detected in DNA sequence analysis using Methprimer site (http://www.urogene.org/methprimer/). (D) Comparison of *CG17352* mRNA levels in the whole body of wild type (*WT*), revertant (*RV*) and *IDH* mutant (*IDH*^*P*^) flies (n = 3). (E) Survival curves of control (*hs*) and *CG17352* overexpressing (*hs>CG17352*) male flies under rotenone treatments (log-rank test: P = 0.241, n = 90 for *hs*; n = 87 for *hs>CG17352*). All life span assays were carried out at 25°C and were repeated at least twice. (F) Life span of adult male flies. The number of surviving flies was counted at the indicated days, and the survival ratios were presented as percentile values (log-rank test: P<0.001, n = 115 for *WT*; n = 120 for *IDH*^*P*^; n = 114 for *RV*). All life span assays were carried out at 25°C and were repeated at least twice. (G) *Hsp22* mRNA level of the indirect flight muscle from fly thoraces (n = 3, Student’s two-tailed t test, **, P<0.01). Data information: If not indicated, significance was determined by one-way ANOVA with Sidak correction (*, P<0.05;**, P<0.01). Error bars indicate SD.
(TIF)

S3 Fig Mitochondrial defects in 30-day-old *IDH* mutants. (A-B) Comparison of the ATP contents in fly thoraces from 3- (A) and 30-day-old (B) revertant (*RV*) and *IDH* mutant (*IDH*^*P*^) males grown on normal media (n = 3). (C-D) Quantification of the mtDNA in fly thoraces from 3- (C) and 30-day-old (D) males grown on normal media (n = 3). Cox I, cytochrome c oxidase subunit I; Cox III, cytochrome c oxidase subunit III; Cyt B, cytochrome b. (E-F) Comparison of climbing ability of 3- (E) and 30-day-old (F) flies grown on normal media (n = 5). Data information: Significance was determined by Student’s two-tailed t test (**, P<0.01; ***, P<0.001; NS, not significant). Error bars indicate SD.
(TIF)

S4 Fig IDH inhibits DA neuronal defects of *DJ-1* mutants under H_2_O_2_ treatments. (A-B) Confocal images (A) and graphs (B) of the average number of DA neurons within DL1, DM, PM, and DL2 clusters of the brains from 6-day-old adult flies after H_2_O_2_ treatments (n = 29 for *elav*; n = 30 for other genotypes). DA neurons were stained with anti-TH antibody (green). Scale bars: 20 μm. Data information: Significance was determined by one-way ANOVA with Sidak correction [*, P<0.05; ***, P<0.001; NS, not significant (P>0.05)]. Error bars indicate SD.
(TIF)

S5 Fig *IDH* mutation has no detrimental effect on the oxidative stress-induced defects in *DJ-1β* null mutants. (A) Survival curves of wild type (*WT*), *DJ-1β* null mutants (*DJ-1β*^*ex54*^), *IDH* mutants (*IDH*^*P*^), and *DJ-1β* and *IDH* double mutants (*IDH*^*P*^ *DJ-1β*^*ex54*^) under rotenone treatment (log-rank test: *DJ-1β*^*ex54*^ VS *WT*: P<0.001; *IDH*^*P*^ VS *WT*: P<0.001; *DJ-1β*^*ex54*^ VS *IDH*^*P*^ *DJ-1β*^*ex54*^: P = 0.8297; n = 90 for *IDH*^*P*^; n = 120 for other genotypes). All life span assays were carried out at 25°C and were repeated at least twice. (B-C) Confocal images (B) and graphs (C) of the average number of DA neurons within DL1, DM, PM, and DL2 clusters of the brains from 6-day-old adult flies after rotenone treatments (n = 30 for each genotypes). DA neurons were stained with anti-TH antibody (green). Scale bars: 20 μm. Data information: Significance was determined by one-way ANOVA with Sidak correction [**, P<0.01; NS, not significant (P>0.05)]. Error bars indicate SD.
(TIF)

S6 Fig IDH cannot rescue *PINK1* mutant phenotypes. (A) Comparison of the ATP contents in fly thoraces from 3-day-old *PINK1* null mutants (*B9*, *da*), IDHm1-expressing *PINK1* null mutants (*B9*, *da>IDHm1*), and IDHc-expressing *PINK1* null mutants (*B9*, *da>IDHc*). *da-GAL4/+* (*da*) flies were used as controls (n = 3). (B) Quantification of mtDNA in fly thoraces from 3-day-old flies (n = 3). (C) Comparison of climbing ability of 3-day-old flies (n = 5). (D-E) Confocal images (D) and graphs (E) of the average number of DA neurons within DL1 clusters of the adult brains from 30-day-old *PINK1* null mutants (*B9*, *TH*), IDHm1-expressing *PINK1* null mutants (*B9*, *TH>IDHm1*), and IDHc-expressing *PINK1* null mutants (*B9*, *TH>IDHc*). *TH-GAL4/+* (*TH*) flies were used as controls. DA neurons were stained with anti-TH antibody (green) (n = 40 for each genotype). Scale bar: 20 μm. Data information: Significance was determined by one-way ANOVA with Sidak correction (***, P<0.001; NS, not significant (P>0.05)). Error bars indicate SD.
(TIF)

S7 Fig Generation of *DJ-1* null SN4741 cell line and the efficiency of *Keap1* siRNA. (A) Immunoblot of DJ-1 in wild type (*WT*) and DJ-1 null (*DJ-1*^*-/-*^) SN4741 cell lines was shown. (B) sgRNA target site and indels were shown for *DJ-1* null SN4741 cell line in the diagram. (C) Comparison of *Keap1* mRNA expression levels upon *Keap1* siRNA transfection.
(TIF)

S8 Fig IDH with cancer-associated mutations cannot rescue *DJ-1* mutant phenotypes under oxidative stress. (A) Comparison of IDH activity in IDHm1 (*hs>IDHm1*)-, IDHm1 R134Q (*hs>IDHm1*^*RQ*^)- or IDHm1 R166K (*hs>IDHm1*^*RK*^)-expressing flies. *hs*-GAL4/+ (*hs*) flies were used as controls. (B) Survival curves of *DJ-1β* null mutants (*hs DJ-1β*^*ex54*^), IDHm1-expressing *DJ-1β* null mutants (*hs>IDHm1 DJ-1β*^*ex54*^), IDHm1 R134Q-expressing *DJ-1β* null mutants (*hs>IDHm1*^*RQ*^ *DJ-1β*^*ex54*^) and IDHm1 R166K-expressing *DJ-1β* null mutants (*hs>IDHm1*^*RK*^ *DJ-1β*^*ex54*^) under rotenone treatments (log-rank test: *hs DJ-1β*^*ex54*^ VS *hs>IDHm1 DJ-1β*^*ex54*^: P<0.001; *hs DJ-1β*^*ex54*^ VS *hs>IDHm1*^*RQ*^ *DJ-1β*^*ex54*^: P = 0.331; *hs DJ-1β*^*ex54*^ VS *hs>IDHm1*^*RK*^ *DJ-1β*^*ex54*^: P = 0.012; n = 120 for each genotype). All life span assays were carried out at 25°C and were repeated at least twice. (C-D) Confocal images (C) and graphs (D) of the average number of DA neurons within DL1 and DM clusters of the adult brains from 6-day-old *DJ-1β* null mutants (*elav DJ-1β*^*ex54*^), IDHm1-expressing *DJ-1β* null mutants (*elav>IDHm1 DJ-1β*^*ex54*^), IDHm1 R134Q-expressing *DJ-1β* null mutants (*elav>IDHm1*^*RQ*^ *DJ-1β*^*ex54*^) and IDHm1 R166K-expressing *DJ-1β* null mutants (*elav>IDHm1*^*RK*^ *DJ-1β*^*ex54*^) under rotenone treatments (n = 30 for each genotype). DA neurons were stained with anti-TH antibody (green). Scale bars: 20 μm. (E-F) Confocal images (E) and graphs (F) of the average number of DA neurons within DL1 and DM clusters of the adult brains from the 6-day-old flies under H_2_O_2_ treatments (n = 30 for each genotype). DA neurons were stained with anti-TH antibody (green). Scale bars: 20 μm. Data information: Significance was determined by one-way ANOVA with Sidak correction (*, P<0.05; **, P<0.01; ***, P<0.001; NS, not significant). Error bars indicate SD.
(TIF)

S1 Table The most enriched biological process gene ontological categories in the differentially expressed genes between oxidative stressed *DJ-1* null and wild type flies. (DOCX)

S2 Table The most enriched molecular function gene ontological categories in the differentially expressed genes between oxidative stressed *DJ-1* null and wild type flies. (DOCX)

S3 Table The statistical analysis of the life span assays. (DOCX)
