# Supplementary figures and images for: Isocitrate protects DJ-1 null dopaminergic cells from oxidative stress through NADP+-dependent isocitrate dehydrogenase (IDH)
Source: PLoS Genet. 2017 Aug 21;13(8):e1006975. doi: 10.1371/journal.pgen.1006975 (PMC5578699; doi:10.1371/journal.pgen.1006975)

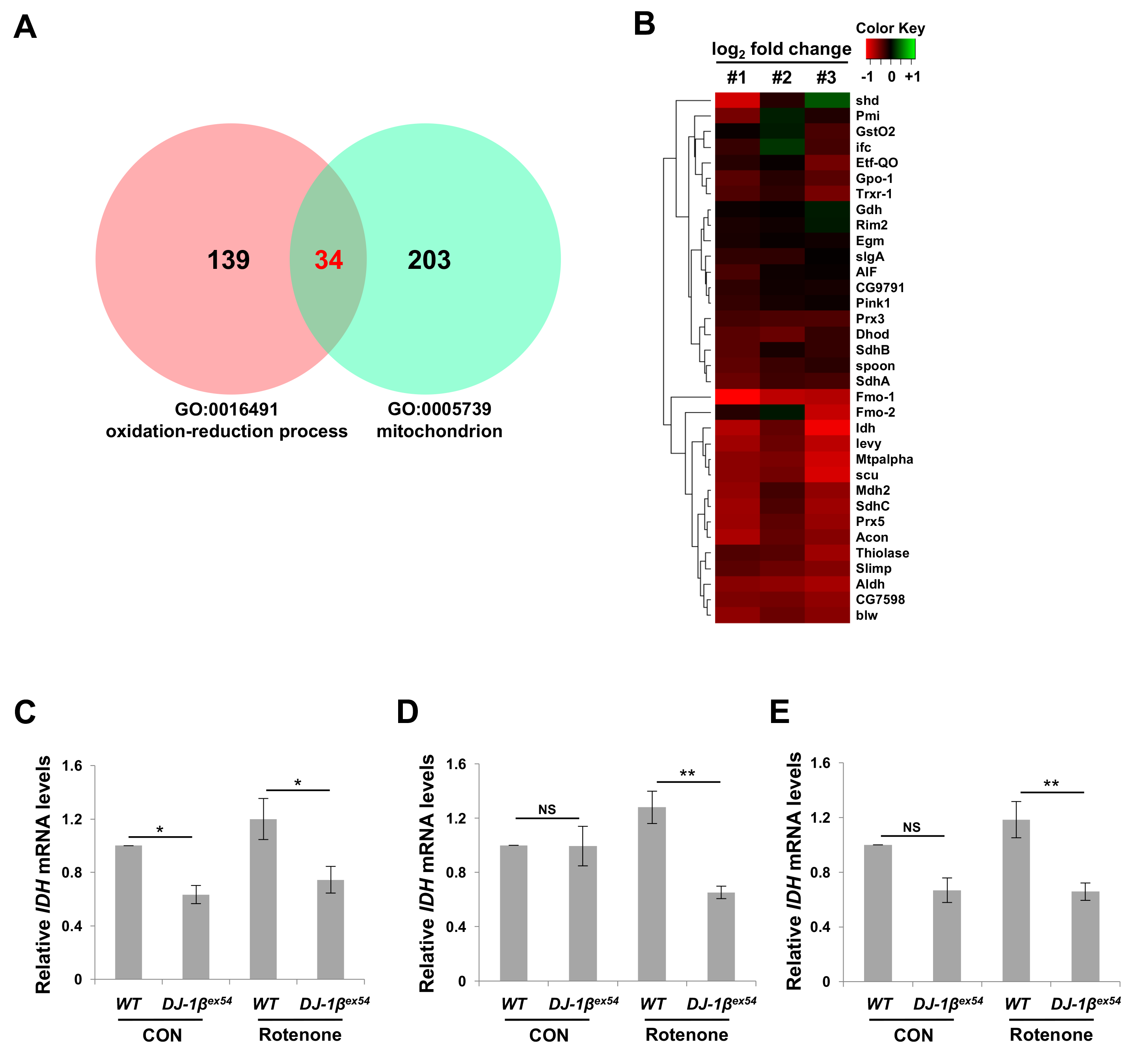

Supplement: S1 Fig — (A) The Venn diagram summarizes the ontology analysis using the genes screened from RNA-seq of wild type and DJ-1β null flies under rotenone treatment. Red circle indicates the number of genes in oxidation-reduction process ontology, and green circle indicates the number of genes in mitochondrion ontology. 34 genes are the ones that fall into both ontologies, reduction-oxidation process and mitochondrion. (B) The heat map presents changes in the expression of the 34 genes mentioned in Fig. S1A. #1, #2, and #3 indicate each of three independent RNA-seq experiments (n = 3). (C-E) Comparison of IDH mRNA levels in heads (C), thoraces (D), and abdomens (E) of wild type flies (WT) and DJ-1β null mutants (DJ-1βex54) under control (CON) or rotenone treatment (Rotenone) (n = 3). (TIF) [file pgen.1006975.s001.tif]

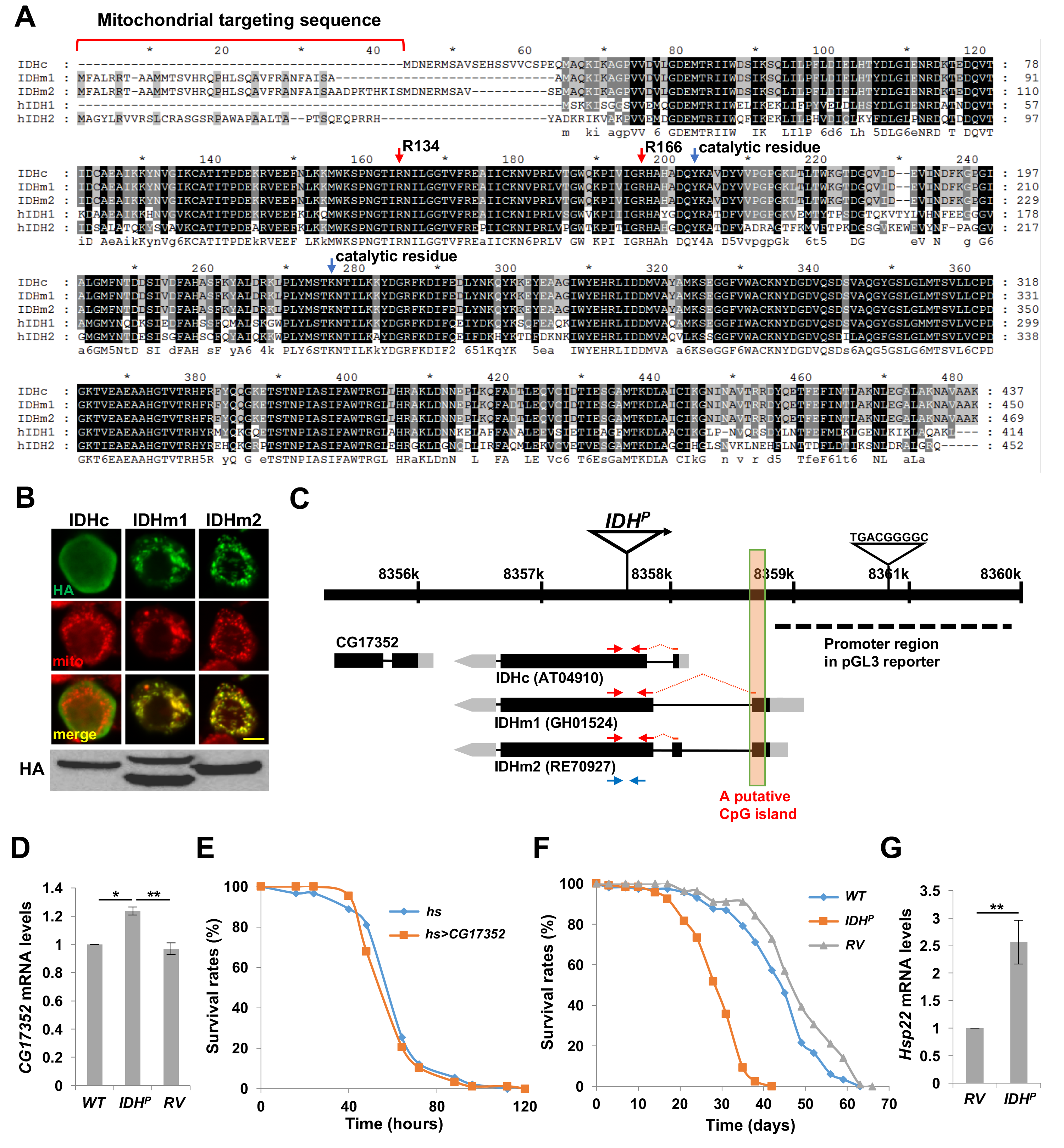

Supplement: S2 Fig — (A) Sequence alignment of Drosophila IDHs (IDHc, IDHm1, and IDHm2), human IDH1 (hIDH1), and human IDH2 (hIDH2). Mitochondrial targeting sequence, catalytic residues, and R134 and R166 residues were indicated. (B) Cytosolic and mitochondrial localization of IDH isoforms. Subcellular localization of C-terminally HA-tagged cytosolic IDH (IDHc) and mitochondrial IDHs (IDHm1 and IDHm2) in S2 cells was determined by co-staining with anti-HA antibody (green) and MitoTracker (red). Anti-HA immunoblots confirmed expression of each isoform. Scale bar: 5 μm. (C) Schematic genomic organization of the IDH locus. Black rectangles: coding sequences (CDS); gray rectangles: untranslated regions (UTR). Genomic structures of IDHP were described in Materials and Methods. The location of the putative Antioxidant Response Element (ARE) (TGACGGGGC) and the promoter region in IDH reporter plasmids were also presented. Binding sites of Quantitative PCR primers for all IDH isoform genes (blue arrows) and each isoform (red arrows) were indicated. Sequences of the primers were described in Materials and Methods. A putative CpG island was detected in DNA sequence analysis using Methprimer site (http://www.urogene.org/methprimer/). (D) Comparison of CG17352 mRNA levels in the whole body of wild type (WT), revertant (RV) and IDH mutant (IDHP) flies (n = 3). (E) Survival curves of control (hs) and CG17352 overexpressing (hs>CG17352) male flies under rotenone treatments (log-rank test: P = 0.241, n = 90 for hs; n = 87 for hs>CG17352). All life span assays were carried out at 25°C and were repeated at least twice. (F) Life span of adult male flies. The number of surviving flies was counted at the indicated days, and the survival ratios were presented as percentile values (log-rank test: P<0.001, n = 115 for WT; n = 120 for IDHP; n = 114 for RV). All life span assays were carried out at 25°C and were repeated at least twice. (G) Hsp22 mRNA level of the indirect flight muscle from fly thoraces (n = 3, [file pgen.1006975.s002.tif]

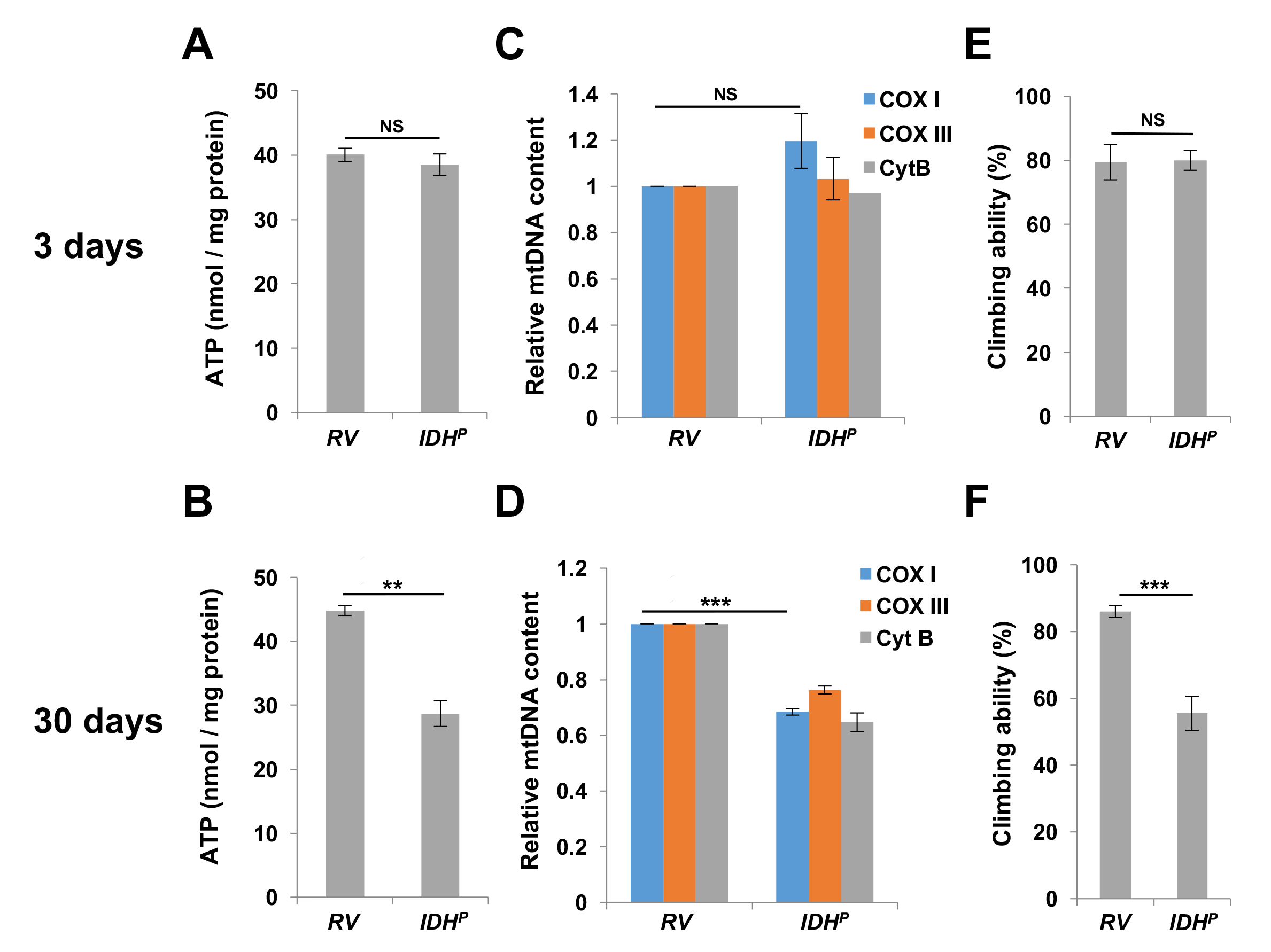

Supplement: S3 Fig — (A-B) Comparison of the ATP contents in fly thoraces from 3- (A) and 30-day-old (B) revertant (RV) and IDH mutant (IDHP) males grown on normal media (n = 3). (C-D) Quantification of the mtDNA in fly thoraces from 3- (C) and 30-day-old (D) males grown on normal media (n = 3). Cox I, cytochrome c oxidase subunit I; Cox III, cytochrome c oxidase subunit III; Cyt B, cytochrome b. (E-F) Comparison of climbing ability of 3- (E) and 30-day-old (F) flies grown on normal media (n = 5). Data information: Significance was determined by Student’s two-tailed t test (**, P<0.01; ***, P<0.001; NS, not significant). Error bars indicate SD. (TIF) [file pgen.1006975.s003.tif]

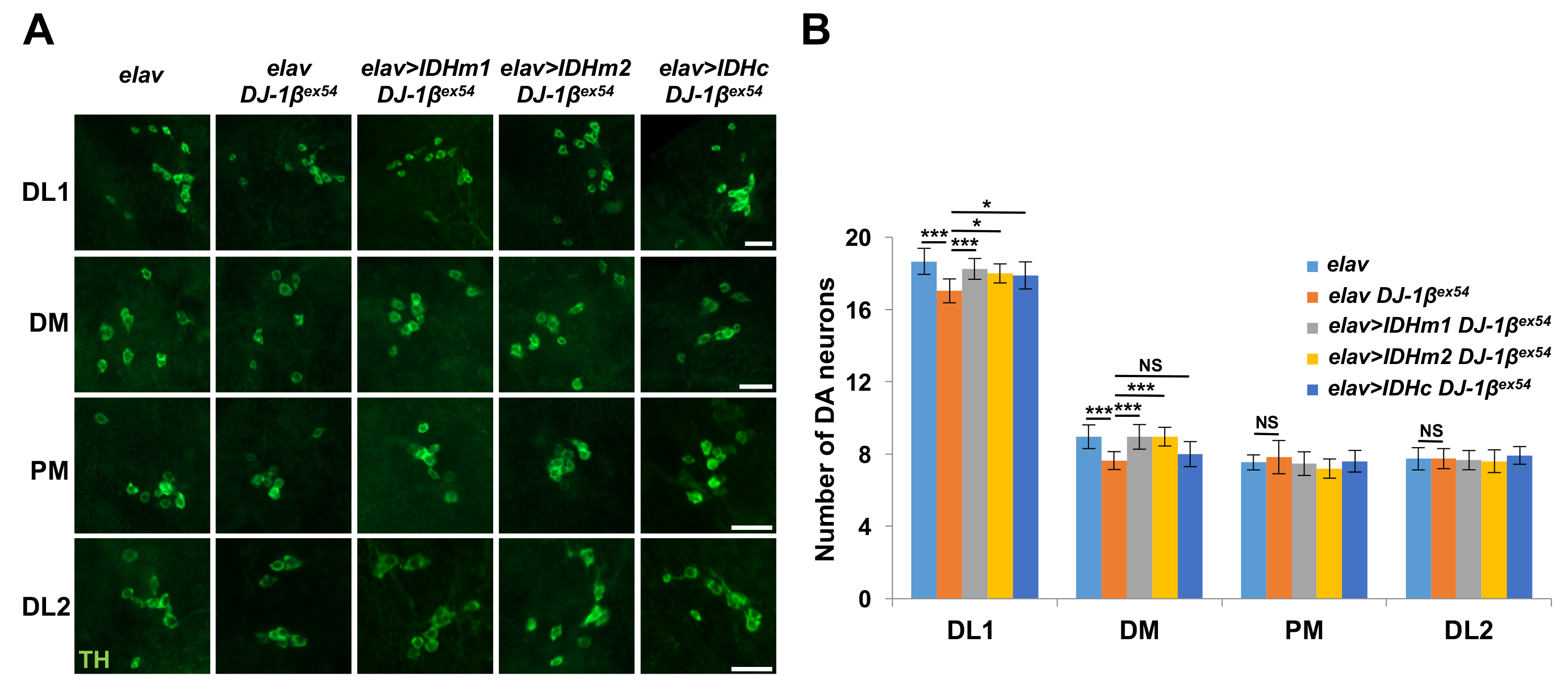

Supplement: S4 Fig — (A-B) Confocal images (A) and graphs (B) of the average number of DA neurons within DL1, DM, PM, and DL2 clusters of the brains from 6-day-old adult flies after H2O2 treatments (n = 29 for elav; n = 30 for other genotypes). DA neurons were stained with anti-TH antibody (green). Scale bars: 20 μm. Data information: Significance was determined by one-way ANOVA with Sidak correction [*, P<0.05; ***, P<0.001; NS, not significant (P>0.05)]. Error bars indicate SD. (TIF) [file pgen.1006975.s004.tif]

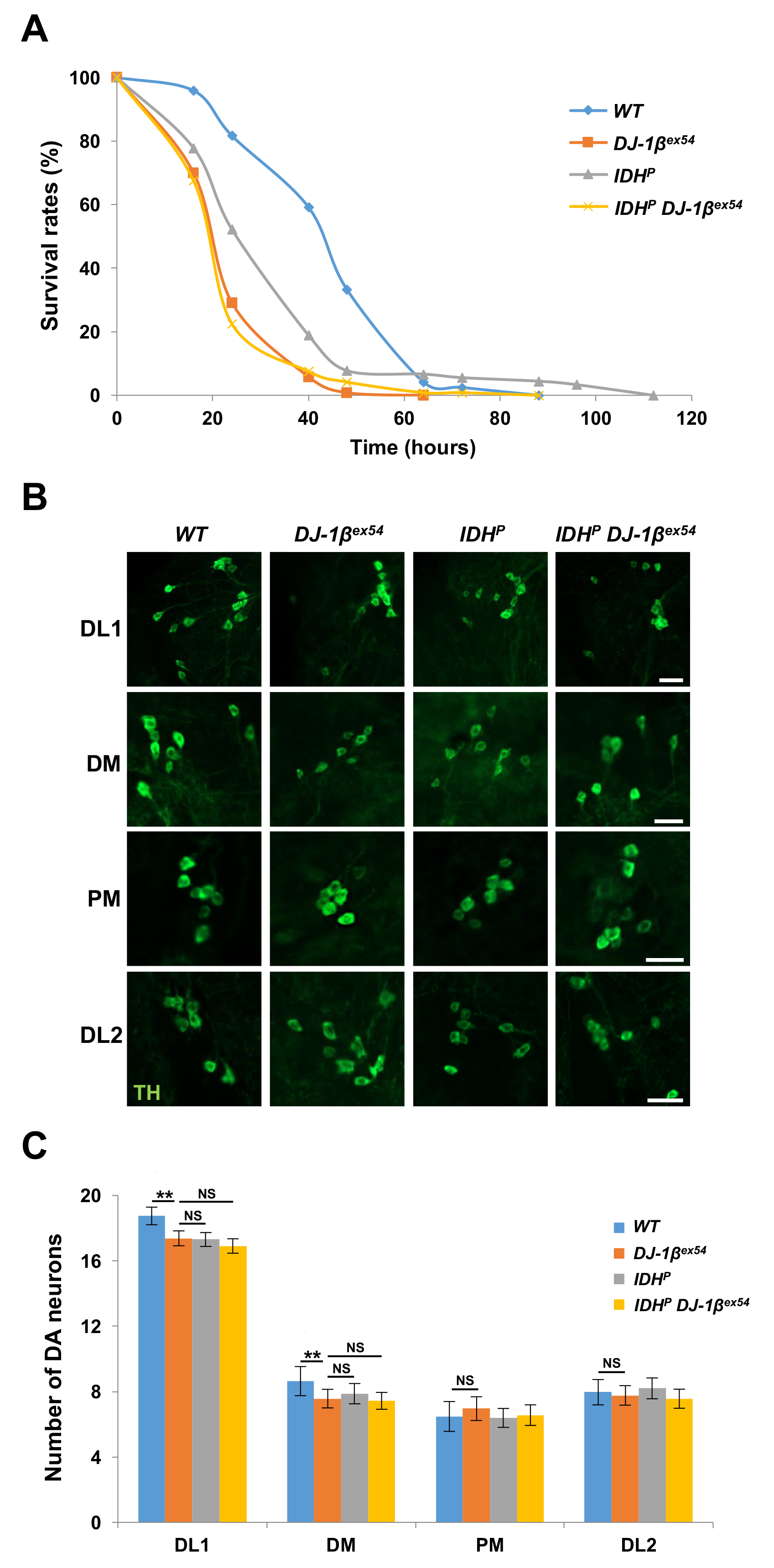

Supplement: S5 Fig — (A) Survival curves of wild type (WT), DJ-1β null mutants (DJ-1βex54), IDH mutants (IDHP), and DJ-1β and IDH double mutants (IDHP DJ-1βex54) under rotenone treatment (log-rank test: DJ-1βex54 VS WT: P<0.001; IDHP VS WT: P<0.001; DJ-1βex54 VS IDHP DJ-1βex54: P = 0.8297; n = 90 for IDHP; n = 120 for other genotypes). All life span assays were carried out at 25°C and were repeated at least twice. (B-C) Confocal images (B) and graphs (C) of the average number of DA neurons within DL1, DM, PM, and DL2 clusters of the brains from 6-day-old adult flies after rotenone treatments (n = 30 for each genotypes). DA neurons were stained with anti-TH antibody (green). Scale bars: 20 μm. Data information: Significance was determined by one-way ANOVA with Sidak correction [**, P<0.01; NS, not significant (P>0.05)]. Error bars indicate SD. (TIF) [file pgen.1006975.s005.tif]

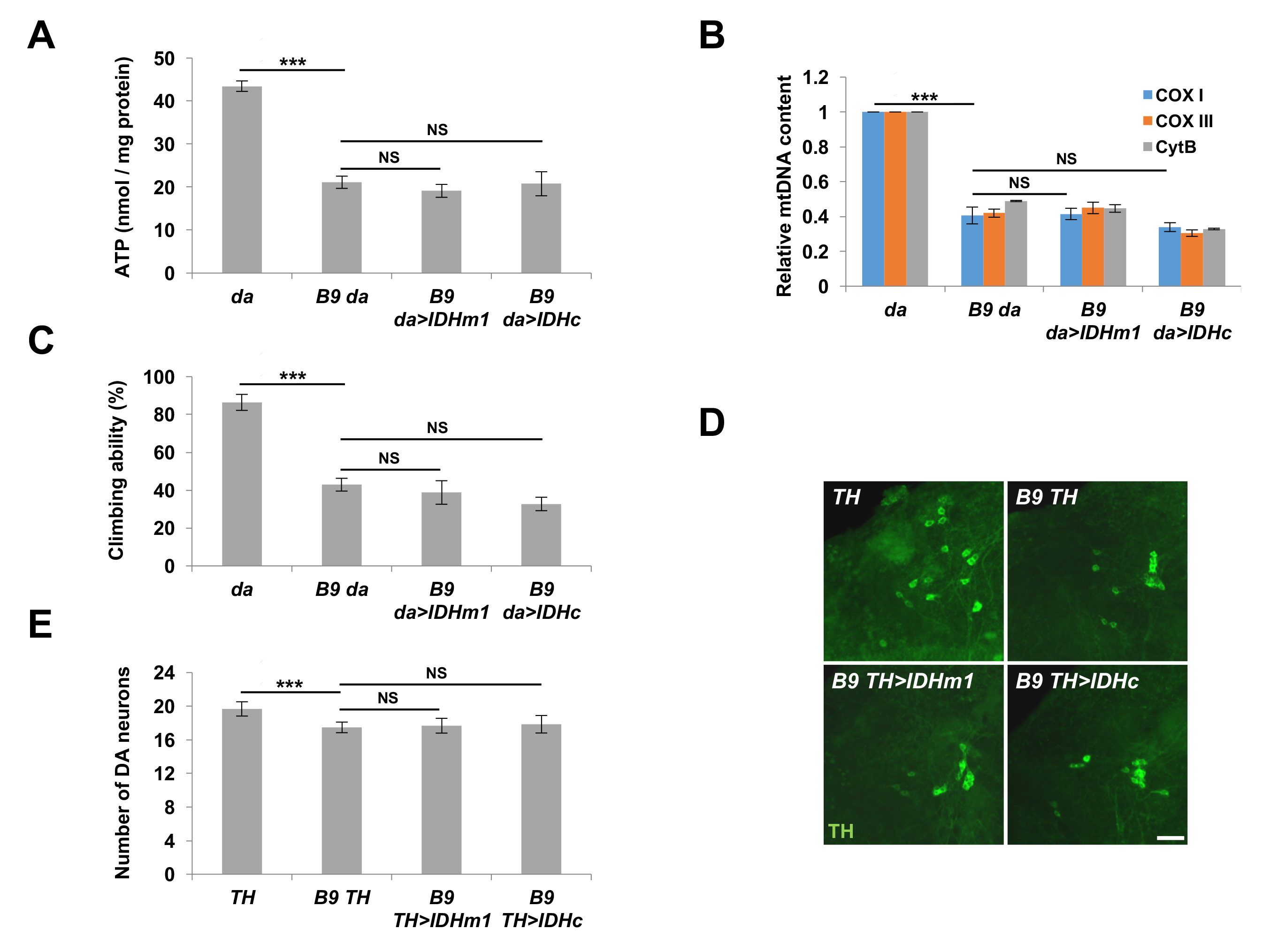

Supplement: S6 Fig — (A) Comparison of the ATP contents in fly thoraces from 3-day-old PINK1 null mutants (B9, da), IDHm1-expressing PINK1 null mutants (B9, da>IDHm1), and IDHc-expressing PINK1 null mutants (B9, da>IDHc). da-GAL4/+ (da) flies were used as controls (n = 3). (B) Quantification of mtDNA in fly thoraces from 3-day-old flies (n = 3). (C) Comparison of climbing ability of 3-day-old flies (n = 5). (D-E) Confocal images (D) and graphs (E) of the average number of DA neurons within DL1 clusters of the adult brains from 30-day-old PINK1 null mutants (B9, TH), IDHm1-expressing PINK1 null mutants (B9, TH>IDHm1), and IDHc-expressing PINK1 null mutants (B9, TH>IDHc). TH-GAL4/+ (TH) flies were used as controls. DA neurons were stained with anti-TH antibody (green) (n = 40 for each genotype). Scale bar: 20 μm. Data information: Significance was determined by one-way ANOVA with Sidak correction (***, P<0.001; NS, not significant (P>0.05)). Error bars indicate SD. (TIF) [file pgen.1006975.s006.tif]

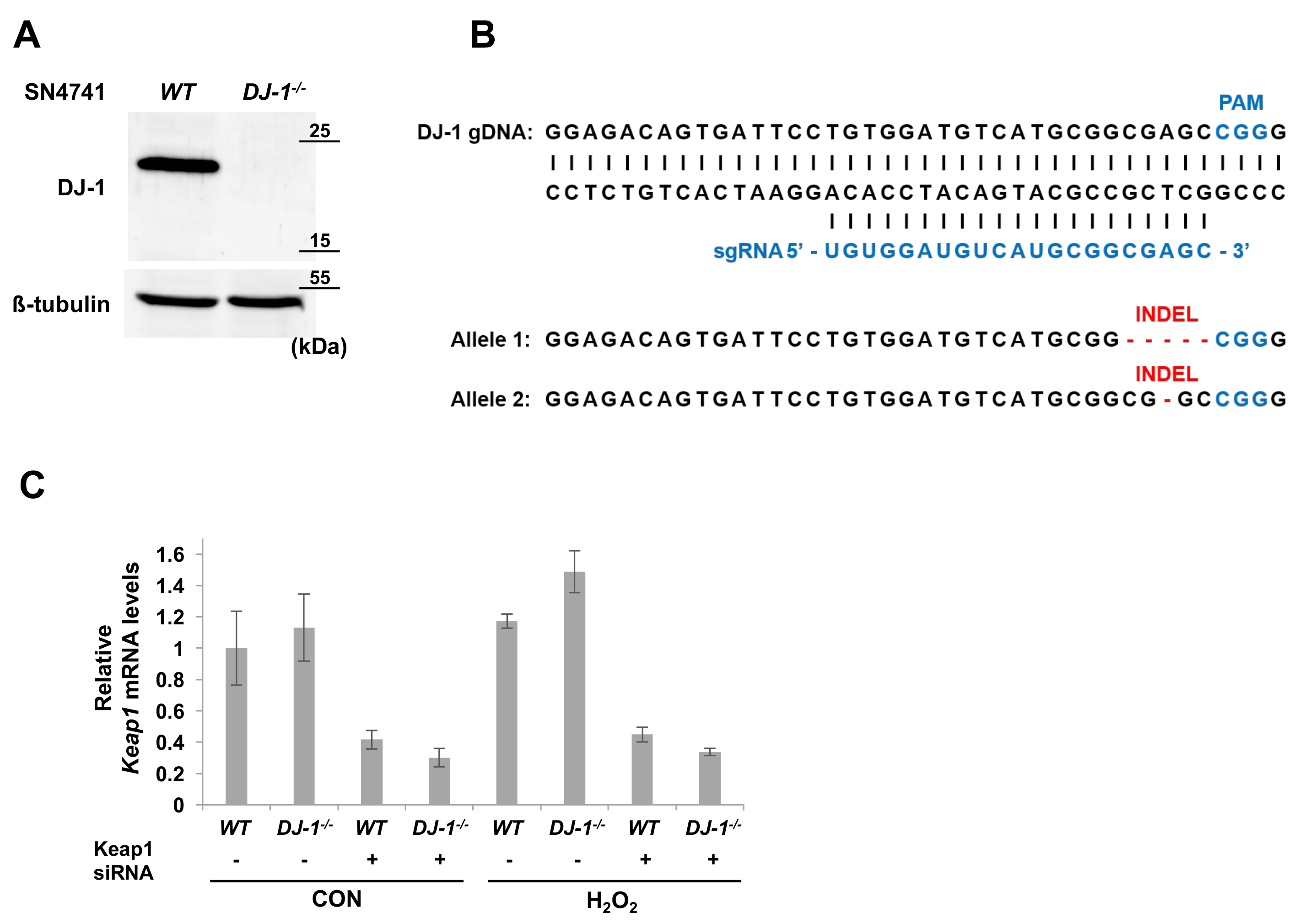

Supplement: S7 Fig — (A) Immunoblot of DJ-1 in wild type (WT) and DJ-1 null (DJ-1-/-) SN4741 cell lines was shown. (B) sgRNA target site and indels were shown for DJ-1 null SN4741 cell line in the diagram. (C) Comparison of Keap1 mRNA expression levels upon Keap1 siRNA transfection. (TIF) [file pgen.1006975.s007.tif]

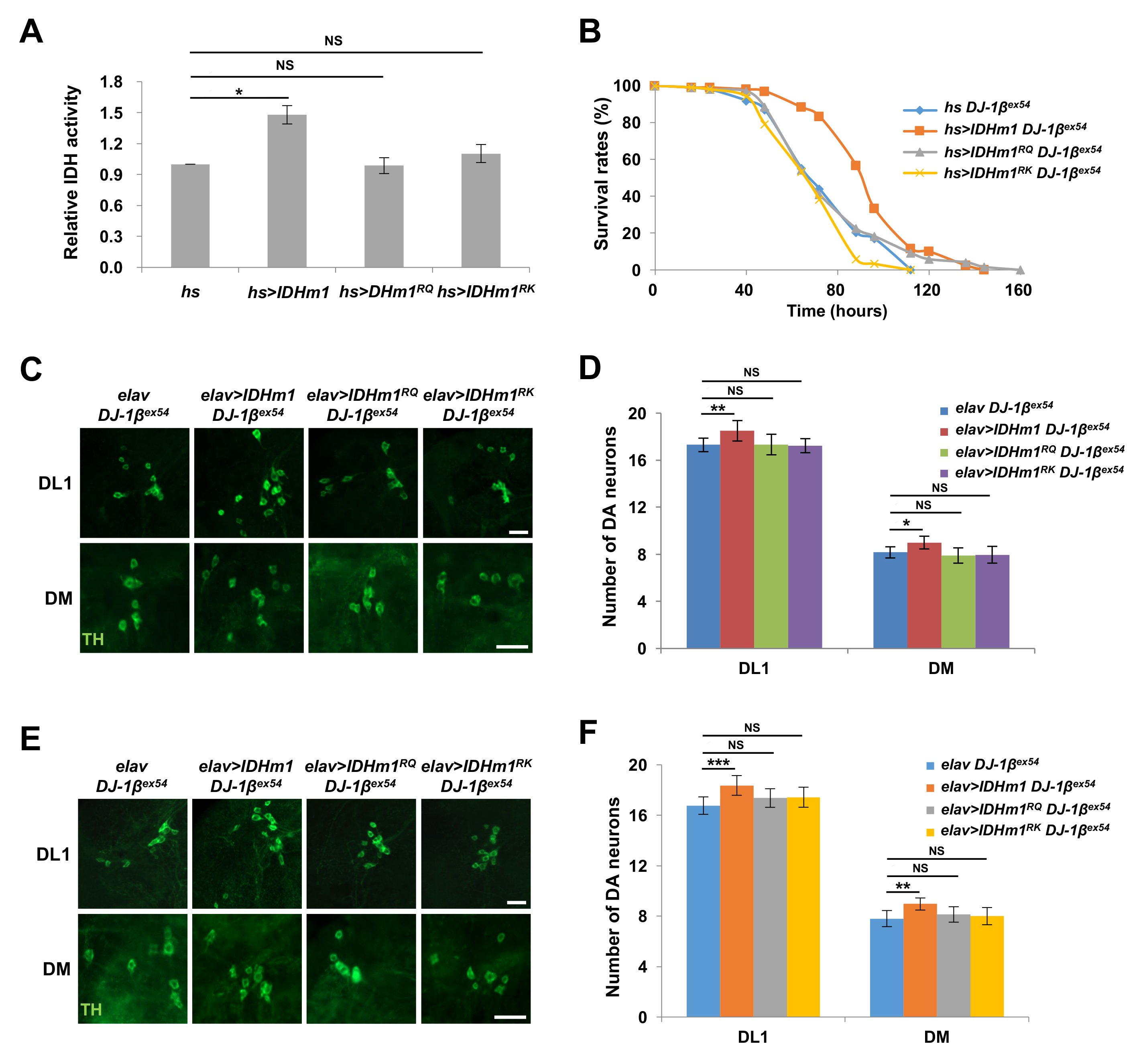

Supplement: S8 Fig — (A) Comparison of IDH activity in IDHm1 (hs>IDHm1)-, IDHm1 R134Q (hs>IDHm1RQ)- or IDHm1 R166K (hs>IDHm1RK)-expressing flies. hs-GAL4/+ (hs) flies were used as controls. (B) Survival curves of DJ-1β null mutants (hs DJ-1βex54), IDHm1-expressing DJ-1β null mutants (hs>IDHm1 DJ-1βex54), IDHm1 R134Q-expressing DJ-1β null mutants (hs>IDHm1RQ DJ-1βex54) and IDHm1 R166K-expressing DJ-1β null mutants (hs>IDHm1RK DJ-1βex54) under rotenone treatments (log-rank test: hs DJ-1βex54 VS hs>IDHm1 DJ-1βex54: P<0.001; hs DJ-1βex54 VS hs>IDHm1RQ DJ-1βex54: P = 0.331; hs DJ-1βex54 VS hs>IDHm1RK DJ-1βex54: P = 0.012; n = 120 for each genotype). All life span assays were carried out at 25°C and were repeated at least twice. (C-D) Confocal images (C) and graphs (D) of the average number of DA neurons within DL1 and DM clusters of the adult brains from 6-day-old DJ-1β null mutants (elav DJ-1βex54), IDHm1-expressing DJ-1β null mutants (elav>IDHm1 DJ-1βex54), IDHm1 R134Q-expressing DJ-1β null mutants (elav>IDHm1RQ DJ-1βex54) and IDHm1 R166K-expressing DJ-1β null mutants (elav>IDHm1RK DJ-1βex54) under rotenone treatments (n = 30 for each genotype). DA neurons were stained with anti-TH antibody (green). Scale bars: 20 μm. (E-F) Confocal images (E) and graphs (F) of the average number of DA neurons within DL1 and DM clusters of the adult brains from the 6-day-old flies under H2O2 treatments (n = 30 for each genotype). DA neurons were stained with anti-TH antibody (green). Scale bars: 20 μm. Data information: Significance was determined by one-way ANOVA with Sidak correction (*, P<0.05; **, P<0.01; ***, P<0.001; NS, not significant). Error bars indicate SD. (TIF) [file pgen.1006975.s008.tif]
