# Supplementary material for: Isocitrate protects DJ-1 null dopaminergic cells from oxidative stress through NADP+-dependent isocitrate dehydrogenase (IDH)
Source: PLoS Genet. 2017 Aug 21;13(8):e1006975. doi: 10.1371/journal.pgen.1006975 (PMC5578699; doi:10.1371/journal.pgen.1006975)
Supplement: S1 Table — (DOCX) [file pgen.1006975.s009.docx]

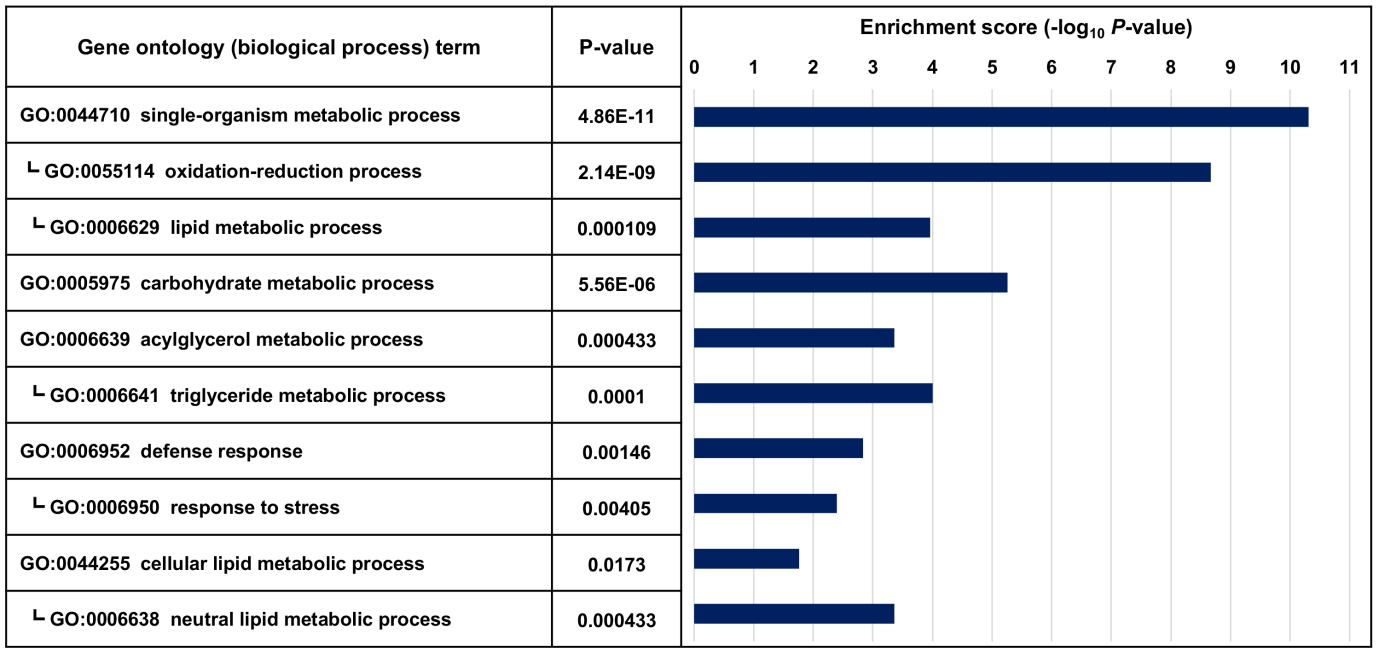


The enrichment score reflects the relative importance of gene ontology terms. The top 10 categories are derived from g:Profiler and Amigo2.
