# Supplementary material for: Isocitrate protects DJ-1 null dopaminergic cells from oxidative stress through NADP+-dependent isocitrate dehydrogenase (IDH)
Source: PLoS Genet. 2017 Aug 21;13(8):e1006975. doi: 10.1371/journal.pgen.1006975 (PMC5578699; doi:10.1371/journal.pgen.1006975)
Supplement: S3 Table — (DOCX) [file pgen.1006975.s011.docx]

| **Genotype** | **Media** | **Mean lifespan (±SD)** | **75% mortality** | **n** | ***P*-value against control** | **Figure** |
| --- | --- | --- | --- | --- | --- | --- |
|  |  |  |  |  |  |  |
| ***WT*** | **normal** | **43.7(±1.05)d** | **49d** | **115** | **<0.001** | **Fig S2E** |
| ***RV*** | **normal** | **48.3(±0.97)d** | **56d** | **114** | **<0.001** | **Fig S2E** |
| ***IDH^P^*** | **normal** | **29.2(±0.64)d** | **31d** | **120** | **-** | **Fig S2E** |
|  |  |  |  |  |  |  |
| ***hs*** | **rotenone** | **64.1(±1.74)h** | **88h** | **90** | **-** | **Fig S2G** |
| ***hs CG17352*** | **rotenone** | **62.3(±1.52)h** | **72h** | **87** | **0.241** | **Fig S2G** |
|  |  |  |  |  |  |  |
| ***RV*♂** | **rotenone** | **88.2(±2.53)h** | **96h** | **90** | **-** | **Fig 1I** |
| ***IDH^P^*♂** | **rotenone** | **67.6(±2.52)h** | **88h** | **90** | **<0.001** | **Fig 1I** |
|  |  |  |  |  |  |  |
| ***RV*♀** | **rotenone** | **86.3(±2.42)h** | **96h** | **90** | **-** | **Fig 1I** |
| ***IDH^P^*♀** | **rotenone** | **70.1(±2.72)h** | **88h** | **90** | **<0.001** | **Fig 1I** |
|  |  |  |  |  |  |  |
| ***hs*** | **rotenone** | **84.9 (±2.36)h** | **96h** | **120** | **<0.001** | **Fig 2A** |
| ***hs DJ-1β^ex54^*** | **rotenone** | **54.1 (±1.36)h** | **64h** | **120** | **-** | **Fig 2A** |
| ***hs IDHm1 DJ-1β^ex54^*** | **rotenone** | **79.0 (±2.55)h** | **96h** | **120** | **<0.001** | **Fig 2A** |
| ***hs IDHm2 DJ-1β^ex54^*** | **rotenone** | **69.8 (±1.81)h** | **88h** | **120** | **<0.001** | **Fig 2A** |
| ***hs IDHc DJ-1β^ex54^*** | **rotenone** | **72.5 (±1.95)h** | **88h** | **120** | **<0.001** | **Fig 2A** |
|  |  |  |  |  |  |  |
| ***WT*** | **rotenone** | **47.5 (±1.48)h** | **64h** | **120** | **<0.001** | **Fig S5A** |
| ***DJ-1β^ex54^*** | **rotenone** | **26.9 (±0.97)h** | **40h** | **120** | **-** | **Fig S5A** |
| ***IDH^P^*** | **rotenone** | **35.6 (±2.19)h** | **40h** | **90** | **<0.001** | **Fig S5A** |
| ***IDH^P^ DJ-1β^ex54^*** | **rotenone** | **26.5 (±1.15)h** | **24h** | **120** | **0.829** | **Fig S5A** |
|  |  |  |  |  |  |  |
| ***WT*** | **rotenone** | **36.1(±1.31)h** | **48h** | **120** | **<0.001** | **Fig 3D** |
| ***DJ-1β^ex54^*** | **rotenone** | **27.8(±0.92)h** | **40h** | **120** | **-** | **Fig 3D** |
| ***Keap1^EY5/+^*** | **rotenone** | **49.6(±1.44)h** | **64h** | **150** | **<0.001** | **Fig 3D** |
| ***DJ-1β^ex54^ Keap1^EY5/+^*** | **rotenone** | **47.5(±2.20)h** | **64h** | **140** | **<0.001** | **Fig 3D** |
|  |  |  |  |  |  |  |
| ***WT*** | **H_2_O_2_** | **92.6(±2.31)h** | **112h** | **109** | **<0.001** | **Fig 3E** |
| ***DJ-1β^ex54^*** | **H_2_O_2_** | **71.1(±1.29)h** | **88h** | **119** | **-** | **Fig 3E** |
| ***Keap1^EY5/+^*** | **H_2_O_2_** | **100.6(±2.72)h** | **112h** | **100** | **<0.001** | **Fig 3E** |
| ***DJ-1β^ex54^ Keap1^EY5/+^*** | **H_2_O_2_** | **96.5(±2.18)h** | **112h** | **120** | **<0.001** | **Fig 3E** |
|  |  |  |  |  |  |  |
| ***hs DJ-1β^ex54^*** | **rotenone** | **77.3(±1.90)h** | **88h** | **123** | **-** | **Fig S8B** |
| ***hs IDHm1 DJ-1β^ex54^*** | **rotenone** | **97.0(±1.84)h** | **112h** | **120** | **<0.001** | **Fig S8B** |
| ***hs IDHm1^RQ^ DJ-1β^ex54^*** | **rotenone** | **79.9(±2.34)h** | **88h** | **120** | **0.331** | **Fig S8B** |
| ***hs IDHm1^RK^ DJ-1β^ex54^*** | **rotenone** | **71.6(±1.62)h** | **88h** | **120** | **0.012** | **Fig S8B** |
